# Whole transcriptome analysis to identify non-coding RNA regulators and hub genes in sperm of non-obstructive azoospermia by microarray, single-cell RNA sequencing, weighted gene co-expression network analysis, and mRNA-miRNA-lncRNA interaction analysis

**DOI:** 10.1186/s12864-024-10506-9

**Published:** 2024-06-11

**Authors:** Danial Hashemi Karoii, Hossein Azizi, Thomas Skutella

**Affiliations:** 1 Department of Cell and Molecular Biology, School of Biology, College of Science, University of Tehran, Tehran, Iran; 2 Faculty of Biotechnology, Amol University of Special Modern Technologies, Amol, Iran; 3 Institute for Anatomy and Cell Biology, Medical Faculty, University of Heidelberg, Im Neuenheimer Feld 307, 69120 Heidelberg, Germany

**Keywords:** Sperm, Non-coding RNA, Infertility, Genes expression, Non-obstructive azoospermia, Single-cell

## Abstract

**Background:**

The issue of male fertility is becoming increasingly common due to genetic differences inherited over generations. Gene expression and evaluation of non-coding RNA (ncRNA), crucial for sperm development, are significant factors. This gene expression can affect sperm motility and, consequently, fertility. Understanding the intricate protein interactions that play essential roles in sperm differentiation and development is vital. This knowledge could lead to more effective treatments and interventions for male infertility.

**Materials and methods:**

Our research aim to identify new and key genes and ncRNA involved in non-obstructive azoospermia (NOA), improving genetic diagnosis and offering more accurate estimates for successful sperm extraction based on an individual’s genotype.

**Results:**

We analyzed the transcript of three NOA patients who tested negative for genetic sperm issues, employing comprehensive genome-wide analysis of approximately 50,000 transcript sequences using microarray technology. This compared gene expression profiles between NOA sperm and normal sperm. We found significant gene expression differences: 150 genes were up-regulated, and 78 genes were down-regulated, along with 24 ncRNAs up-regulated and 13 ncRNAs down-regulated compared to normal conditions. By cross-referencing our results with a single-cell genomics database, we identified overexpressed biological process terms in differentially expressed genes, such as “protein localization to endosomes” and “xenobiotic transport.” Overrepresented molecular function terms in up-regulated genes included “voltage-gated calcium channel activity,” “growth hormone-releasing hormone receptor activity,” and “sialic acid transmembrane transporter activity.” Analysis revealed nine hub genes associated with NOA sperm: *RPL34, CYB5B, GOL6A6, LSM1, ARL4A, DHX57, STARD9, HSP90B1*, and *VPS36*.

**Conclusions:**

These genes and their interacting proteins may play a role in the pathophysiology of germ cell abnormalities and infertility.

**Supplementary Information:**

The online version contains supplementary material available at 10.1186/s12864-024-10506-9.

## Introduction

Male infertility and sterility have long been linked to sperm abnormalities in the majority of animals investigated [1]. These abnormalities range from obvious morphological defects to those that are more subtly faulty on clinical assessment [2]. In general, sperm shape may influence both fertilization and pregnancy outcomes. Environmental, genetic, or a combination of both factors may contribute to defective sperm shape. Although environmental reasons are thought to be the most frequent, there is a growing list of sperm structural defects thought to be genetic [3].

Even though bull fertility heritability is typically thought to be low, some factors of bull fertility, such as sperm morphological defects, are under genetic influence [4]. Inbreeding is associated with increasing proportions of defective seminiferous tubules in humans, according to other research. It was found that frozen semen decreased the adverse effects of genital health and semen quality by 62% (even though AI bulls were previously evaluated for genital health and semen quality) [5, 6].

An estimated 16% of couples experience infertility, and approximately a third of the time, the male is the primary cause [7]. Many factors contribute to male infertility, including infertility, varicocele, infections of the reproductive system, endocrine disorders, obstructive azoospermia (OA), and non-obstructive azoospermia (NOA) [8, 9]. One of the most common causes of male infertility, NOA affects 1% of males and accounts for 10–15% of infertile men [10, 11]. Infertility in men due to spermatogenic dysfunction of the testicles is called NOA. Patients with NOA either cannot produce or only a small amount of Sperm [12]. This condition refers to a situation where there is a lack of sperm in the ejaculate due to a problem with sperm production in the testes. It’s a significant concern for couples facing infertility issues [13]. Highlighting the importance of NOA in reproductive medicine underscores the challenges many couples encounter when trying to conceive. NOA necessitates specialized diagnostic techniques and treatments, such as testicular sperm extraction (TESE) or microdissection TESE (micro-TESE), to retrieve viable sperm for assisted reproductive techniques like in vitro fertilization (IVF) or intracytoplasmic sperm injection (ICSI) [14]. Addressing NOA effectively requires a multidisciplinary approach involving reproductive endocrinologists, urologists, embryologists, and genetic counselors. It also underscores the importance of ongoing research to advance our understanding of male infertility and improve treatment outcomes for affected individuals and couples.

Genetic defects can play a significant role in male infertility, particularly in cases of NOA, where sperm production is impaired within the testes. For example, Certain regions of the Y chromosome are crucial for sperm production [15]. Microdeletions in these regions can lead to NOA. For instance, the AZF (azoospermia factor) regions on the Y chromosome are known to be associated with spermatogenesis. Deletions in these regions can impair sperm production. In addition, chromosomal disorder occurs when a male is born with an extra X chromosome (XXY instead of XY). Klinefelter syndrome is a common cause of male infertility and is associated with reduced testicular function, leading to NOA. Mutations in the CFTR gene, associated with cystic fibrosis, can also impact male fertility [16]. These mutations can cause congenital bilateral absence of the vas deferens (CBAVD), a condition where the tubes that carry sperm from the testes to the urethra are absent. CBAVD often leads to obstructive azoospermia, but in some cases, it can also result in NOA due to impaired sperm production [17, 18].

Spermatogenic cell maturation and meiosis are impaired in NOA patients due to altered structure of the testicular seminiferous tubules [19]. A recent study has shown that the spermatogenesis problem involves both localized and diverse organs [20]. Several mutations genes have recently been identified by whole-exome sequencing in NOA pedigrees, including *Dynein axonemal assembly factor 2 (DNAAF2)* [21], *leucine-rich repeat-containing 6 (LRRC6)* [22], and *PIH domain containing 3 (PIH1D3)* [23], *sperm flagellar protein 2 (Spef2)* [24], *septin (SEPT)* [25], *testis anion transporter 1 (TAT1)* [26], *DnaJ homolog subfamily B member 13 (DNAJB13)* and *sperm mitochondria-associated cysteine-rich protein* (SMCP) [26]. This study finds sperm gene and ncRNA expression deficiencies that may explain the two hallmarks of the human non-obstructive azoospermia testis mentioned above. Anti-Müllerian Hormone (AMH) is a gene that codes for a protein involved in the regulation of ovarian follicle development. Its expression levels can provide insights into ovarian reserve and follicular development, which are critical for female fertility assessment [27]. Follicle-Stimulating Hormone Receptor (FSHR) is a gene that encodes the receptor for follicle-stimulating hormone (FSH), a key regulator of ovarian function and follicle maturation in females [28]. Variations in FSHR gene expression can affect ovarian response to fertility treatments such as controlled ovarian stimulation. Androgen Receptor (AR) gene codes for the androgen receptor, which mediates the effects of testosterone and other androgens. Androgens play essential roles in male reproductive development, spermatogenesis, and fertility. Dysregulation of AR expression can contribute to male infertility [29]. Anti-Müllerian Hormone Receptor Type 2 (AMHR2) is the gene that encodes the receptor for AMH. It is involved in the signaling pathway that regulates Müllerian duct regression during male fetal development and follicle recruitment in females. Variations in AMHR2 expression may impact reproductive outcomes [30]. Estrogen Receptor Alpha (ESR1) is a gene that codes for the estrogen receptor alpha, which mediates the effects of estrogen in reproductive tissues. Estrogen signaling is crucial for various aspects of female reproductive health, including menstrual cycle regulation, endometrial function, and fertility [31]. Inhibin Alpha Subunit (INHA) gene encodes the alpha subunit of inhibin, a hormone that regulates FSH secretion from the pituitary gland. Inhibin plays a role in feedback regulation of follicle development and ovarian function in females. Progesterone Receptor (PR) is a gene that codes for the progesterone receptor, which mediates the effects of progesterone in the female reproductive system. Progesterone signaling is essential for preparing the endometrium for implantation and maintaining early pregnancy [32].

Zhang et al. [33] identified and characterized circular RNAs in testicular tissue of NOA patients, implicating their potential role in disease pathogenesis. Similarly, Ji et al. [34] demonstrated the predictive value of testis-derived circular RNAs in seminal plasma for the outcome of microdissection testicular sperm extraction in idiopathic NOA. These findings underscore the relevance of spermatic circRNAs as diagnostic and prognostic markers in NOA. Moreover, Manfrevola et al. [35] elucidated the role of circRNAs and the circRNA-dependent network (ceRNET) in asthenozoospermia, highlighting the broader implications of RNA-mediated regulatory networks in male infertility. Furthermore, studies by Sabetian et al. [36], Bo et al. [37], Song et al. [38], and Lian et al. [39] have delineated dysregulated mRNA-miRNA-lncRNA interactions, altered expression profiles of long noncoding RNAs, and microRNAs associated with NOA, providing crucial insights into the molecular landscape of this condition.This results in mechanisms unique to post-meiotic male germ cells controlling gene expression (morphological remodeling involving extensive chromatin condensation, reduced nuclear and cytoplasmic volume, formation of an acrosome system and tail, etc.) [40–43]. The activator of CREM in testis (ACT) is a sperm transcriptional coactivator that controls cAMP response element modulator (CREM) activity. ACT modulates CREM activity via Kif17b, a kinesin specific to germ cells that regulates its distribution within cells. Moreover, CREM-targeted mRNA is transported and stabilized by sperm RNA-binding proteins. It is critical to male fertility that post-meiotic sperm exhibit this extensive and complex control of gene expression [44–47].

Single-cell analysis offers a detailed view of molecular mechanisms in various diseases with remarkable precision. This approach encompasses diverse techniques like single-cell RNA sequencing (scRNA-seq), single-cell assay for transposase-accessible chromatin sequencing (scATAC-seq), and spatial transcriptomics. Each technique provides unique insights: scRNA-seq has unveiled previously unknown cell types in the testis that aid in spermatogenesis. Meanwhile, scATAC-seq, which examines open chromatin at the individual cell level, has been instrumental in identifying crucial transcription factors active during meiosis. Spatial transcriptomics adds another dimension by integrating spatial information, addressing the gap in tissue architecture context that other single-cell analyses might miss. This comprehensive toolkit allows for a more nuanced understanding of cellular function and disease progression at the single-cell level like male and female infertility. During in vitro fertility, whole-genome sequencing was not required in some studies examining stage association in the human seminiferous epithelium. Additionally, whole-genome sequencing has been studied in male germ cells. Using a microarray and an in-silico analysis, we showed and analyzed the expression of whole genome sequencing genes in sperm.

## Materials and methods

### Ethics and consent

This investigation was carried out in 2016 using testicular material from three adult male patients with various medical backgrounds and three healthy individuals. All human material tests undertaken here were authorized by the local ethical committees Heidelberg (Ethics Committee of the Medical Faculty of Heidelberg University) and Amol University of Special Modern Technologies (Iran National Committee for Ethics in Biomedical Research: approved number: Ir.ausmt.rec.1402.05) and informed written consent was provided by all human participants.

### Patient and control selection

**Fig. 1:**
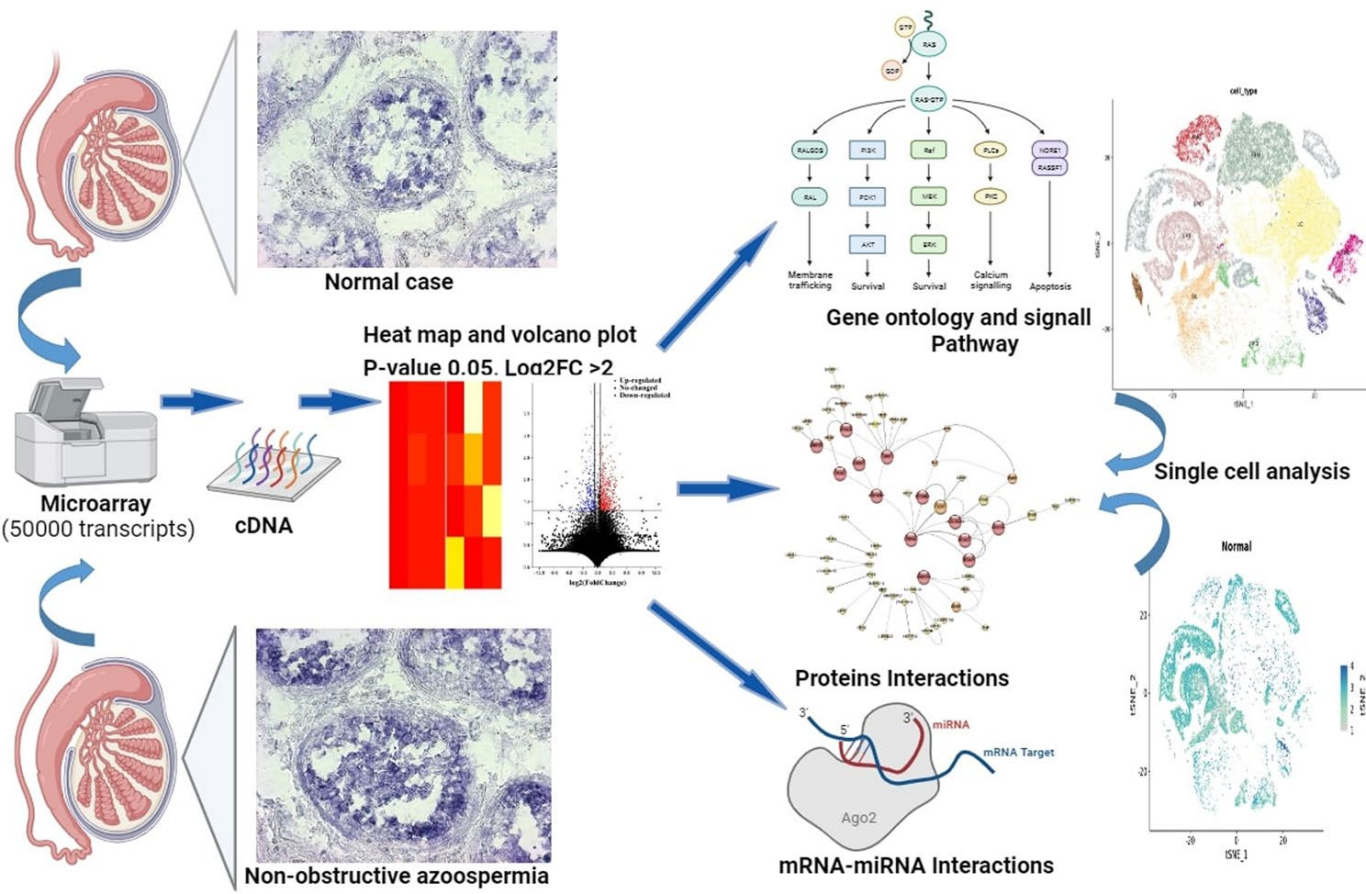
Diagram of the experimental design

Three instances of azoospermia caused by faulty spermatogenesis and three control cases were examined in this investigation (Table 1; Fig. 1). Following a testicular biopsy for genetic testing, an oocyte intracytoplasmic injection was arranged. Oocyte retrieval and ICSI cycles, 4–5 months later, did not correspond with testicular biopsies and sperm extraction. Testicular spermatozoa were cryopreserved. The Heidelberg University Ethics Committee authorized the initiative. Serum samples were collected and analyzed according to WHO guidelines. Azospermia was identified after 12 min of 1500 g centrifugation of semen without spermatozoa. Urological operations, gonadotoxic exposure, developmental, social, medical, and reproductive histories were considered. Each patient had a general, systemic, and genital exam. Testicular volume was determined using a Prader orchidometer. This research measured plasma LH and FSH levels using enzyme-linked immunoassays (normal values are 2–3 IU/I and 3–10 IU/I, respectively). Plasma testosterone (9.4–37.0 nmol/l) was measured by radioimmunoassay. Assay coefficients of variation were limited to 6.5%. Before testicular biopsy, all venous blood samples were chromosomal analyzed. Y-chromosome microdeletion tests were not performed on all cases. After suspecting inadequate sperm volume, acidic pH, or vasa deferens deficiency, CF gene mutation cascade screening was undertaken to rule out obstructive azoospermia. Patients with retrograde ejaculation, endocrine problems, and genital tract obstructions were excluded. Only patients with spermatogenesis-related azoospermia were studied [48–53].

**Table 1:** Characteristics of men including in the program of in vitro fertilization providing sperm samples analyzed in this study

Patient (Sample)	Indication	Histopathological diagnosis	Source of Sperm	Sperm quality	Fertilization	Pregnancy
Patient 1	non-obstructive azoospermia	Hypospermatogenesis	Testis	Concentration: 60 × 10^6^ spermatozoa/ml Motility: 70% motile Morphology: 31% normal	+	**-**
Patient 2	non-obstructive azoospermia	Hypospermatogenesis	Testis	Concentration: 200 × 10^6^ spermatozoa/ml Motility: 50% motile Morphology: 48% normal	+	**-**
Patient 3	non-obstructive azoospermia	Hypospermatogenesis	Testis	Concentration: 80 × 10^6^ spermatozoa/ml Motility: 70% motile Morphology: 26% normal	+	**-**
Normal	Normozoospermia	/	Ejaculate	Abundant, motile spermatozoa	+	+
Normal	Normozoospermia	/	Ejaculate	Abundant, motile spermatozoa	+	+
Normal	Normozoospermia	/	Ejaculate	Abundant, motile spermatozoa	+	-

To provide more detail on the patient selection process and criteria for selecting patients with non-obstructive azoospermia and healthy controls, we meticulously considered several factors. Firstly, patients with non-obstructive azoospermia were selected based on a comprehensive evaluation of their reproductive health history, including urological operations, gonadotoxic exposure, and developmental, social, medical, and reproductive backgrounds. Additionally, each patient underwent a thorough general, systemic, and genital examination, including testicular volume determination using a Prader orchidometer. Furthermore, specific laboratory tests were conducted to confirm the diagnosis, such as semen analysis following centrifugation and hormonal assays measuring plasma LH, FSH, and testosterone levels. Chromosomal analysis of venous blood samples was performed before testicular biopsy to rule out any chromosomal abnormalities. While Y-chromosome microdeletion tests were not performed on all cases, suspicion of inadequate sperm volume, acidic pH, or vasa deferens deficiency prompted CF gene mutation cascade screening to exclude obstructive azoospermia.

In contrast, healthy controls were carefully selected to ensure they were unaffected by factors that could influence the results. These controls underwent a similar evaluation process, including comprehensive medical history assessments and physical examinations to confirm their reproductive health. Exclusion criteria were applied to eliminate individuals with retrograde ejaculation, endocrine problems, or genital tract obstructions. Moreover, serum samples were collected and analyzed according to WHO guidelines to confirm their reproductive health status.

Overall, the patient selection process involved a multidimensional approach, incorporating detailed medical histories, comprehensive physical examinations, and rigorous laboratory testing to ensure accurate categorization of both non-obstructive azoospermia patients and healthy controls [45, 54–57].

### Surgical and sample processing

Transverse incisions were made in the albuginea of the testis, either equatorially or in the cranial section, to remove a substantial quantity of testicular tissue. After washing the pieces in a human tubal fluid medium, the biologist examined the samples under a microscope. Afterwards, gentamicin 75 mg/100 ml Ringer solution was irrigated onto the testicular tissue surfaces for antisepsis. As a final step, we used gauze moistened with antiseptic solution to push the testicular tissue for 3 min, followed by very restricted bipolar micro coagulation. After 1.5 mg betamethasone was injected into the vaginal cavity, a continuous vicryl 5/0 was applied to the tunica vaginalis opening to avoid discomfort and adhesions. Using glass slides, we separated and minced individual seminiferous tubules from the testicular tissue and placed them in sterile Petri plates with 0.75 ml of a sperm-washing medium. To determine whether or not spermatozoa were present in each tissue sample, each sample was counted. We assessed the viability of the extracted spermatozoa by eosin-nigrosin staining when the re-covered fluid contained more than 100 spermatozoa/mm3. In the appendix, you’ll find a description of this study’s experimental design (Table 2). In the first phase of the study, gene expression patterns on microarrays were analysed from a variety of cell types.

**Table 2:** Related GEO dataset information of microarray and single cell of NOA.

GSE	NOA	Normal	platform
GSE45885	**27**	**3**	**Microarray (HuGene-1_0-st)**
GSE9210	**47**	**10**	**Microarray (Agilent-012097 Human 1 A)**
GSE108886	**8**	**4**	**Microarray (Illumina HumanHT-12 V4.0)**
GSE145467	**10**	**10**	**Microarray (Agilent-014850 Whole Human Genome Microarray 4 × 44 K G4112F)**
GSE216907	**8**	**6**	**RNA-seq (Illumina HiSeq 2000)**
GSE235324	**50**	**50**	**RNA-seq (Illumina NovaSeq 6000)**

### cDNA microarrays

Positive phage clones were randomly selected from a human testis l phage cDNA library (Hl5503U, Clontech, Palo Alto, Calif.) and amplified using polymerase chain reaction (PCR). As previously disclosed, the PCR products were subsequently spotted onto membranes to create human testis cDNA microarrays [58].

### Collection of human semen samples

Healthy participants provided human ejaculates. A routine sperm analysis was done in accordance with WHO guidelines from 2016. Sperm were classed as normal or motility-impaired based on this research (Table 1). Normal Sperm has > 20,106 spermatozoa/ml with more than 50% active sperm, more than 25% of the sperm moving vigorously in one direction (rapid and linearly progressive), and 1 lymphocyte per high power field. 39 normal sperm samples were obtained in total. Ten of them were homogenized, and utilized in experiment 1 to hybridize with human testis cDNA microarrays. Another 29 normal sperm samples were treated independently and utilized in experiment 2. Semen samples were also taken from 24 patients with poor sperm motility, defined as fewer than 40% active sperms, 5% of which moved quickly in a linear progression, and 1 lymphocyte per high power field (40). Table 1 lists the parameters for the sperm samples. After allowing the sperm samples to liquefy at room temperature, they were washed twice in phosphate-buffered saline (0.1 M PBS).

### Labelling probes and preparing RNA

Isolation of sperm RNA from sperm samples was performed according to manufacturer’s instructions using Trizol RNA isolation reagent (Gibco BRL, Grand Island, USA). Compared to normal sperm motility and sperm motility affected individuals, ejaculated sperm from ten healthy individuals was examined. Using gel electrophoresis and UV spectrophotometry, RNA preparations were assessed for quality and quantity. In order to make the cDNA probes, 33P-labeled dATP was added to a reverse transcription solution containing 50 mg of total spermatozoal RNA, oligo(dT), M-MLV reverse transcriptase, and 200 mCi of alpha-33PdATP (NEN Life Science, Boston, Mass.).

### RNA extraction and hybridization of cDNA microarrays from the testis

A global early maturation arrest pattern was seen in NOA patients’ testicles. TWO sperm samples showed azoospermia. We cannot collect testicular samples from participants with known fertility, which would be ideal for normal control studies. Urological patients without meiotic problems or infertility were investigated, and histology showed normal spermatogenesis. None of the controls received hormonal adjuvant therapy before orchiectomy.

Testicular tissues were fast-frozen in liquid nitrogen and stored at -80 °C until processing. Total RNA was isolated using Trizol (Invitrogen, USA) per manufacturer’s instructions. UV absorbance and denaturing agarose gel electrophoresis assessed isolated RNA quality. cDNA microarrays were pre-incubated at 68 C for 3 h with pre-hybridization solution (6SSC, 0.5% SDS, 5Denhardt’s, 100 mg/ml denatured salmon sperm DNA) and hybridized for 20 h with 33Plabeled human spermatozoa cDNA probes. Before phosphor screening overnight, the microarray membranes were washed with wash solution (0.1SSC, 0.5% SDS) for 1 h at 65 degrees Celsius. In the Fuji Photo Film FLA-3000 A plate/fluorescent image analyzer, the membrane was analyzed. Using 65,536 grayscale pixels of 50 mm, Fuji Photo Film’s Array Gauge software linearly scanned each point’s radioactive intensity. After removing background from places without PCR products, the software determined positive clones as those having signal intensities over 10. A standard deviation of 0.3 indicated appropriate signal intensities from two dots in each DNA sample.

### Microarray analysis

Qiagen’s RNeasy Mini Kit was used to extract RNA and Ambion’s MessageAmp aRNA Kit was used to amplify the extracted RNA from sperm. RNA direct lysis solution was added to each sample immediately after harvesting 200 cells per probe, and kept at -75 °C for 10 l. At the Microarray Facility of the University of Tübingen Hospital, the samples were tested. Analyzing gene expression was done using the Affymetrix Human U133 + 2.0 Genome Oligonucleotide Array. Normalization and biostatistical analysis were performed by MicroDiscovery GmbH of Berlin, Germany.

### Differential expression analysis and data processing

In non-obstructive azoospermia, 50,000 transcripts were evaluated using a microarray. A comparison is then made between these genes and those found in normal cells. |log 1.5 fold change (log2FC) | > 1.5 and adj. value 0.05 are the default settings. Differential expression data can be visualized using volcano maps and heat maps. The volcanic map and heat map were created using the R software.

### Sorting and comparing groups of proteins

Comparing differentially expressed genes between the three research groups was done using the online program ArrayMining. A gene ontology analysis tool, PANTHER (http://www.pantherdb.org/), was used to analyze the gene list.

### Investigating pathways enriched in gene ontology (GO)

A tool called Enrich, an online tool dedicated to functional gene annotation (http://amp.pharm.mssm.edu/Enrichr/), was used to analyze the KEGG and Reactome enrichment pathways. We conducted functional gene enrichment analyses using the STRING enrichment analysis in Cytoscape software to confirm the biological roles of the genes involved in the protein-protein interaction network of the first protein-protein interaction (PPI) node with the RNA sequence. According to the ShinyGO tool, related infertility genes mediate biological processes.

### Network of PPIs for target genes

In order to identify further associations between candidate target genes, the STRING database (https://string-db.org/) and Cytoscape program (https://cytoscape.org/) are used. As a measure of node size, CytoHubba uses the degree value. Genes up-and-down-regulated by the plug-in MCC algorithm are selected. Additionally, the STRING database integrates diverse sources of biological data, including genomic context, co-expression, and curated pathways, to comprehensively annotate protein-protein interactions. Cytoscape, on the other hand, offers a wide range of plugins and algorithms for network analysis, allowing for the visualization and exploration of complex biological networks beyond simple PPIs. Furthermore, CytoHubba employs various algorithms beyond degree centrality, such as betweenness centrality and closeness centrality, to assess the importance of nodes within a network. In our study, the use of CytoHubba’s diverse node ranking algorithms provided a multi-faceted perspective on the regulatory landscape, enhancing the robustness of our findings.

### Identification of lncRNA target genes involved NOA

To find proteins associated with type 2 diabetes mellitus in Homo sapiens, the NCBI database was used (https://www.ncbi.nlm.nih.gov). The target genes for LncRNA NRONs were predicted using NPInter (http://bigdata.ibp.ac.cn/npinter4), RNAInter (http://www.rnainter.org), LncRNADisease (http://www.rnanut.net/lncrnadisease), and RAID databases (https://www.rna-society.org/raid2). We then compared the predicted targets for NRON with the proteins involved in NOA, and visualized their regulatory networks using Cytoscape [59] (version 3.9.1). These databases offer a wealth of information on RNA-protein interactions, including binding affinities, interaction domains, and functional annotations, enabling a comprehensive exploration of lncRNA-mediated regulatory mechanisms. By integrating predictions from multiple databases, we sought to minimize false positives and enhance the reliability of our target gene predictions. Moreover, the MCC algorithm is particularly effective in identifying densely connected regions within a network, thus pinpointing key regulatory hubs that may play pivotal roles in disease pathogenesis. By leveraging the MCC algorithm, we aimed to capture not only individual genes but also interconnected gene modules that collectively orchestrate complex biological processes.

### Profiling of lncRNA expression using array star human

The Human LncRNA Array V3.0 was utilized to profile lncRNA expression, as previously reported [60, 61]. To hybridize with the Human LncRNA Array V3.0 (8660 K; Arraystar), RNA samples from NOA and healthy cases were filtered to eliminate rRNA before being transcribed into fluorescent cRNA probes. According to the most reputable databases (such as RefSeq and Ensembl) and relevant literature, the array comprises 22,520 lncRNAs. Array results were compared to control samples using MultiExperiment Viewer software to see if lncRNA expression increased or decreased. Upregulation was defined as a twofold rise, and downregulation as a twofold decrease. Furthermore, the integration of multiple databases allowed for cross-validation of predicted target genes, thereby reinforcing the robustness of our findings. Through the visual representation of regulatory networks in Cytoscape, we aimed to provide an intuitive framework for understanding the intricate interplay between lncRNAs and their target genes in the context of NOA. Moreover, the utilization of established databases such as RefSeq and Ensembl ensured the accuracy and completeness of our lncRNA annotation, facilitating the interpretation of expression data in a biologically meaningful context. By imposing stringent fold-change criteria for differential expression analysis, we aimed to focus on lncRNAs with the most pronounced alterations in expression levels, thereby prioritizing candidates with potential functional significance.

### Construction of the lncRNA network and topological analysis

The Pearson’s correlation coefficients (PCCs) between each DELs-DEMs pair in oral cancer were calculated to identify lncRNA-miRNA-mRNA interactions as potential ceRNA triples. The DEL-DEM pairs were considered codysregulated if their PCC values ranked in the top 0.05 percentile (PCC > 0.886) with a p-value 0.05. This lncRNA-miRNA-mRNA interaction would then be identified as a dysregulated competing triple after confirmation that both mRNA and lncRNA were targeted by the same miRNA. With Cytoscape 3.9.1, we visualized the regulation network built from the interactions between lncRNAs, miRNAs, and mRNAs in order to gain insight into their roles in the ceRNA network. Data sets with complex topologies require topological analysis to reveal information. Node degree and betweenness centrality (BC), both network topological features, were computed to study the geometric relationships between the data nodes. As a result, the hub nodes in oral spermatogensis regulation networks were considered to be those with a more than five-connection node degree and a higher BC value. Additionally, the identification of hub nodes based on both degree and betweenness centrality metrics provided a nuanced understanding of network topology, highlighting not only highly connected nodes but also those serving as crucial bridges between disparate network modules. Through this comprehensive topological analysis, we aimed to uncover key regulatory nodes driving dysregulated pathways in oral spermatogenesis, shedding light on potential therapeutic targets for intervention.

### lncRNA-mRNA co-expression network

A lncRNA-mRNA co-expression network was constructed to correlate lncRNAs with direct regulated expression of target mRNAs. An algorithm was quoted from a previous report. In order to construct the network, we calculated Pearson correlations for each pair of genes and then selected the pairs with significant correlations (0.98 or greater). Cytoscape was used to draw the co-expression networks. An edge indicates a strong correlation between two genes, and each gene corresponds to a node in this representation. Our core degree calculation was used to determine the relative significance of a gene or lncRNA within the network by counting the number of genes or lncRNAs that are directly linked to each other. The bigger the degree it has, the more significant it is. The normalized degrees of nodes were calculated according to their co-expression networks, and the differences reflect differences in the importance of expression of their corresponding mRNAs/lncRNAs. Degrees are normalized by dividing their degrees by their maximum degrees. A node’s normalized degree of a node is compared between a control and a transformed co-expression network to determine the rank of essential mRNA or lncRNA. To detect significant correlations between lncRNAs and mRNAs analyzed, Pearson correlation tests were used. Only Pearson’s correlation coefficients ≥ 0.9 (*p* < 0.01) were used to construct the network and generate visual representations. Furthermore, the calculation of Pearson correlation coefficients allowed for the quantification of co-expression strength between lncRNAs and target mRNAs, providing insights into the regulatory relationships underlying gene expression dynamics. By comparing co-expression patterns between control and transformed networks, we aimed to identify dysregulated interactions driving aberrant gene expression in oral cancer, thereby elucidating novel mechanisms underlying disease progression.

### Weighted gene co-expression network analysis (WGCNA)

The complex WGCNA approach was used to discover gene co-expression modules linked to hub gene co-expression. Gene co-expression networks have been generated using the WGCNA R package based on NOA and control DEGs [62]. Briefly, genes with comparable expression patterns were assigned to a co-expression module using a weighted correlation adjacency matrix and cluster analysis. To create a weighted adjacency matrix and fulfill scale-free network conditions, a suitable soft threshold β was computed. The weighted adjacency matrix was subsequently converted into a topological overlap matrix (TOM), and the associated dissimilarity was calculated (1-TOM). The dynamic tree cutting technique was used for module identification, and modules with dissimilarities of less than 0.3 were merged. The link between module eigengene values and immune cell abundance was assessed using Pearson correlation. Modules that were strongly correlated with the great majority of gene co-expression were considered critical signaling pathway-related modules and selected for further research.

### Data collection of scRNA-seq

The Male Health Atlas (MHA) version one encompasses nine objects, including two species (Homo sapiens and Mus musculus), five organs/tissues (testis, epididymis, vas deferens, corpus cavernosum, and prostate), eight cell types, and 258,428 unique scRNA data profiles (GSE216907 and GSE235324). It features datasets like the Human Testis Development Atlas, with testicular samples from different ages, and the Human Germ Cell Lineage Atlas, focusing on germ cell lineage from spermatogonial stem cells to spermatids, both with detailed classifications. Additionally, the Human Testis NOA Atlas includes samples from NOA patients and those with normal spermatogenesis, categorized by etiology. Data processing involved the limma and Seurat R packages for bulk RNA-seq and scRNA-seq, respectively, with various methods for cell filtration, normalization, dimensionality reduction, and clustering, including the use of canonical-correlation analysis (CCA) for batch effect elimination. Quality control led to the retention of 61,622 cells from 68,066, with UMAP and resolution selected for further analysis. Cell type annotation utilized marker genes from prior research. The scATAC-seq data used the 10X Genomics Cell Ranger ATAC pipeline and Signac package, with strict quality standards yielding 750 cells. Annotation of scATAC-seq cell types was based on scRNA-seq cell types using the FindTransferAnchors function. Spatial transcriptome data, processed with the Seurat package, were visualized after filtering based on measured genes and unique molecular identifiers (UMIs) (Table 2 and Supplementary 3).

### Data integration and analysis for scRNA-seq

To effectively integrate data from different donors and various scRNA-seq technologies, an anchor-based strategy was employed (GSE45885, GSE9210, GSE108886, GSE145467, GSE216907 and GSE235324). This approach utilizes an unsupervised method to identify a set of anchors, representing a shared biological state, which facilitates data integration. In this process, we utilized 35 dimensions for anchor weighting. Subsequently, the integrated dataset underwent a linear transformation (scaling) and dimensional reduction via principal component analysis (PCA). For visualization and exploration of the integrated dataset, the UMAP method was applied as a non-linear dimensional reduction technique. The number of dimensions for UMAP was determined to be 35, based on the ranking of principal components according to their variance contribution. Default settings were applied for other UMAP parameters. Cell clustering was performed using the graph-based clustering method provided in the Seurat R package. The dimensionality and resolution parameters for constructing a shared nearest neighbor graph and for clustering were set at 35 and 0.3, respectively. Specific markers for testicular germ and somatic cells, identified from existing literature, were utilized to categorize cell types within clusters (Supplementary 3).

### Prediction of miRNA-mRNA target genes and their regulatory networks

To identify the targets of differentially expressed genes and miRNAs, TargetScan (https://www.targetscan.org/vert80/), miRTarBase (https://mirtarbase.cuhk.edu.cn/), and StarBase databases (https://starbase.sysu.edu.cn/) were used. In order to identify candidate target miRNAs, look for the intersection of all three databases and differential genes on microarrays. The regulatory connections between miRNAs and mRNAs are used to construct miRNA-mRNA networks. Cytoscape was used to visualize and find hub signaling pathway of the miRNA-mRNA regulation network.

## Results

### Microarray analysis of gene expression in NOA versus normal cases

**Fig. 2:**
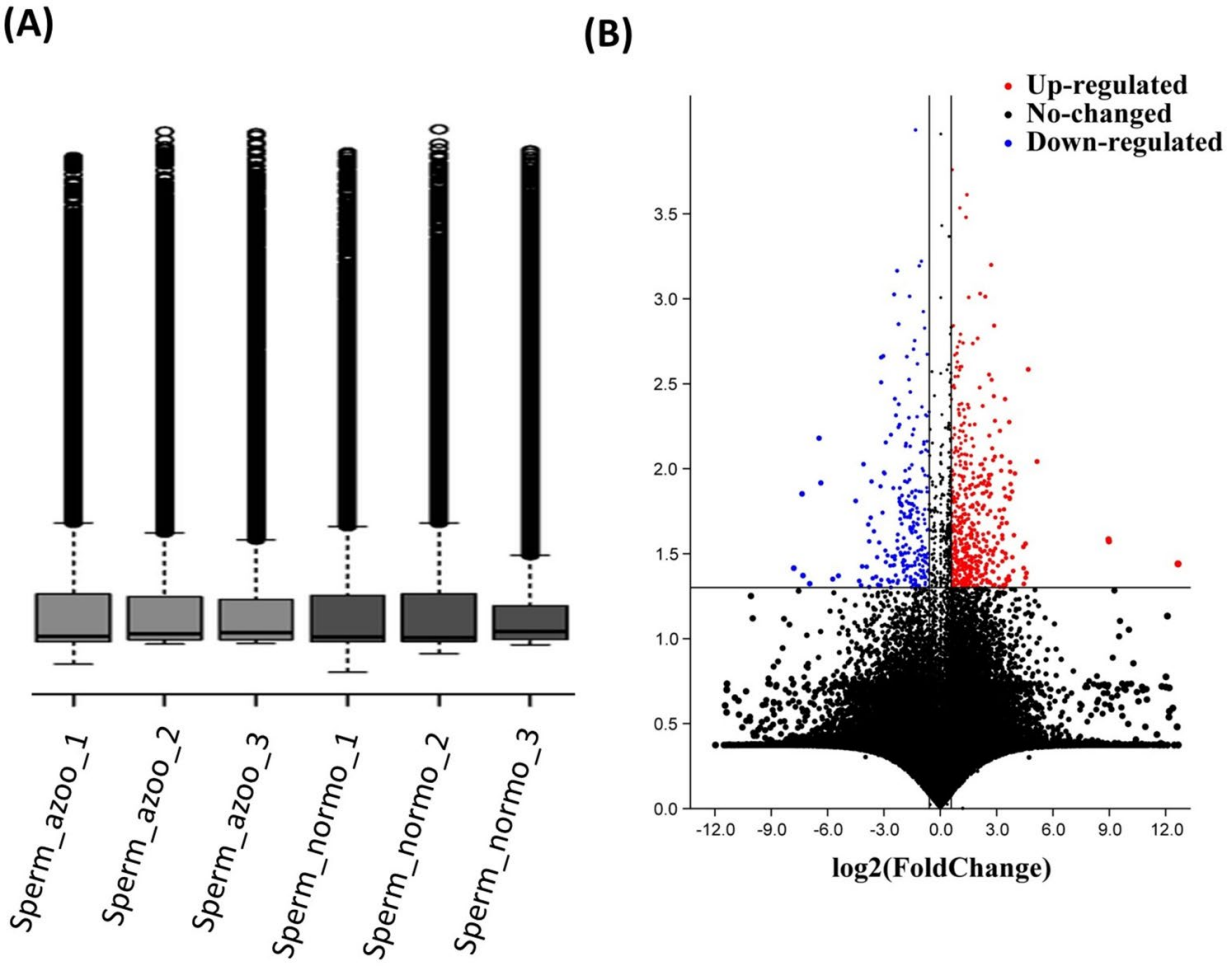
Testis samples with and without non-obstructive azoospermia showed different expressions of genes. (**A**) The normalization of microarray data, and (**B**) the volcano map of the differentially expressed genes. Grey dots indicate no differential expression, red dots indicate upregulation, and blue dots indicate downregulation

**Fig. 3:**
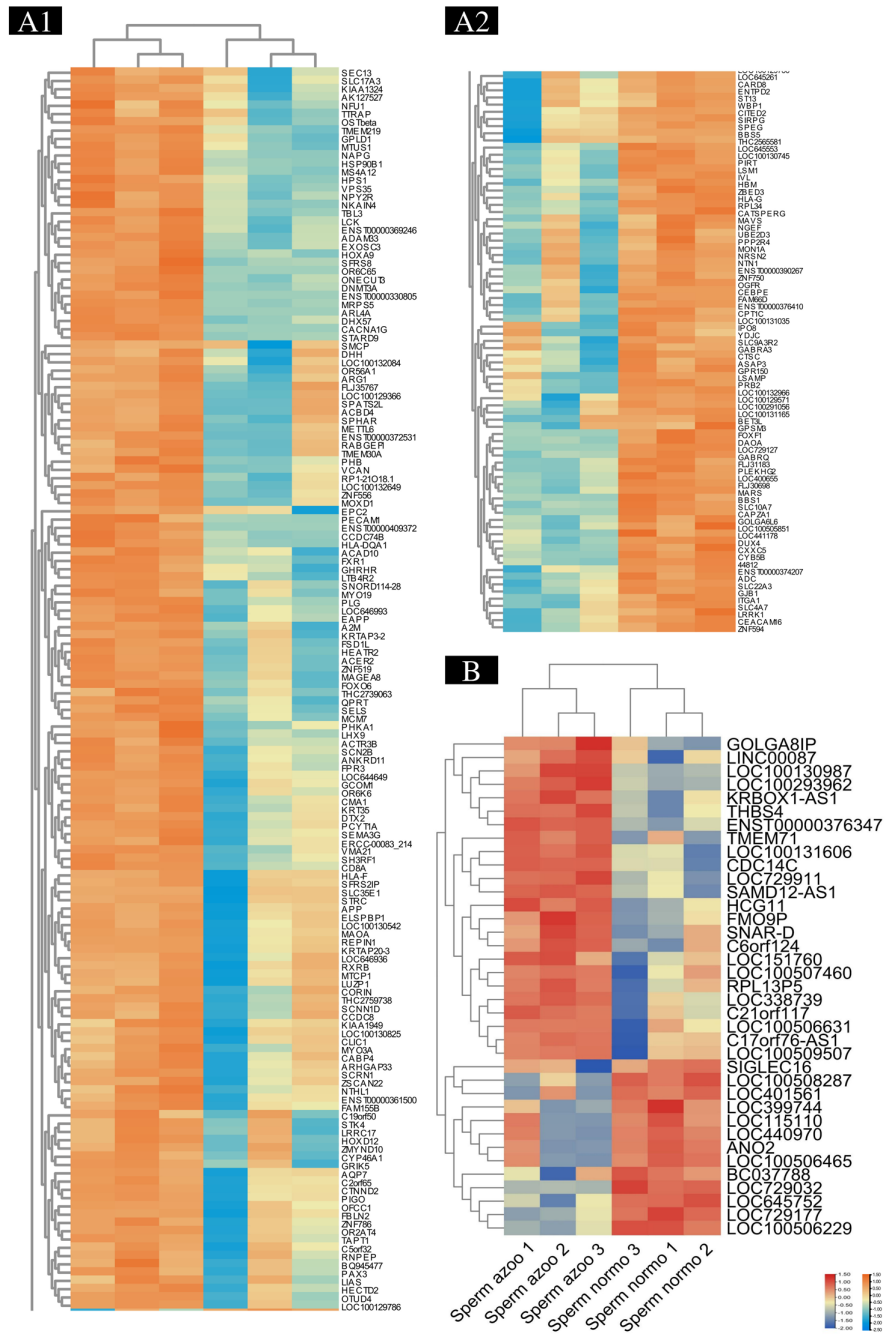
Genes and ncRNA with differential expression are shown on a heat map. In the heat map below, (A1) up differentially expressed genes are shown, (A2) down differentially expressed genes are shown and (B) differentially expressed non-coding RNAs are shown. Red and orange represents upregulation, and blue represents downregulation

A microarray was used to analyze the Whole-transcriptome (about 50,000). Using microarray analysis, we identified 150 genes that were up-regulated and 78 genes that were down-regulated in three non-obstructive azoospermia cases (Figs. 2 and 3). The microarray analysis of three human cases with different non-obstructive azoospermia revealed that *RPL34, DUX4, CYB5B, GOLGA6L6, LSM1, SLC22A3, ADC, MARS, PIRT, HBM, PLEKHG2, SLC10A7, FAM66D, NRSN2, CATSPERG, FOXF1, SLC9A3R2, LSAMP, CXXC5, DAOA, ASAP3, ANO2, GABRQ, CAPZA1, SIRPG, OGFR, CPT1C, BBS1, IVL, GJB1, BC037788, CEBPE, UBE2D3, GPR150, HLA-G, MON1A, BBS5, ZNF594, CITED2, BET3L, ITGA1, ST13, SPEG, CARD8, CTSC, IPO8, SIGLEC16, GABRA3, ZNF750, WBP1, NTN1, PPP2R4, ZBED3, SLC4A7, MAVS, LRRK1, GPSM3, NGEF, ENTPD2, YDJC and PRB2* were down-regulated versus the normal case (Fig. 2).The microarray analysis of three human cases with different NOA showed that *MTCP1, CTNND2, DHH, PECAM1, FMO9P, HECTD2, TTRAP, NFU1, GOLGA8IP, RXRB, SCRN1, PHB, LHX9, OTUD4, SPATS2L, LIAS, THBS4, SLC35E1, HOXA9, KIAA1949, Sect. 13, CLIC1, HOXD12, CABP4, OR2AT4, PHKA1, A2M, STK4, KIAA1324, REPIN1, STRC, CYP46A1, ZSCAN22, SCNN1D, SNAR-D, ACBD4, OR56A1, SEMA3G, ZMYND10, LUZP1, ZNF556, CORIN, ACTR3B, HLA-F, SFRS2IP, MYO3A, NTHL1, LRRC17, RNPEP, CCDC8, APP, ARHGAP33, SH3RF1, SFRS8, HCG11, RABGEF1, SMCP, OFCC1, SNORD114-28, KRTAP20-3, FBLN2, EPC2, SLC17A3, GCOM1, MYO19, THC2759738, ACER2, ADAM33, SPHAR, SELS, MAOA, THC2739063, ELSPBP1, PAX3, PIGO, LCK, TMEM219, EXOSC3, TMEM30A, VCAN, CMA1, QPRT, NPY2R, GHRHR, TMEM71, VMA21, GPLD1, LTB4R2, METTL6, MTUS1, FAM155B, KRT35, AQP7, ARG1, GRIK5, SCN2B, TAPT1, FSD1L, OR6K6, RPL13P5, CDC14C, NAPG, HPS1, NKAIN4, OR6C65, DTX2, PCYT1A, CD8A, MCM7, ACAD10, KRBOX1-AS1, FXR1, ANKRD11, CACNA1G, ZNF519 MAGEA8, TBL3, ONECUT3, HEATR2, PLG, CCDC74B, EAPP, VPS35, SAMD12-AS1, MOXD1, HSP90B1, FPR3, STARD9, FOXO6, HLA-DQA1, DNMT3A, MS4A12, MRPS5, DHX57 and ARL4A* were up-regulated versus the normal case (Supplementary 1 and Fig. 3).

### Microarray comparison of non-coding RNA (ncRNA) expression between NOA and normal cases

Our microarray analysis of whole sequencing (approximately 50,000 transcripts) was performed. As shown in Figs. 1 and 2, the expression of 13 ncRNAs was down-regulated compared to the normal case in three human cases with different NOAs. The analysis of three human cases with different non-obstructive azoospermia revealed that *golgi autoantigen, golgin subfamily a, 6 pseudogene (LOC645752), 60 S ribosomal protein L36-like (LOC729032), LOC100508287, BC037788 (LOC100128946), LOC401561, LOC729177, LOC100506465, LOC100506229, LOC440970, sialic acid binding Ig-like lectin 16 (gene/pseudogene) (SIGLEC16), LOC115110* and *LOC399744* were down-regulated versus the normal case.

The microarray analysis of three human cases with different NOA revealed that *flavin containing monooxygenase 9 pseudogene (FMO9P), LOC100130825, golgin A8 family, member I, pseudogene (GOLGA8IP), small ILF3/NF90-associated RNA D (SNAR-D), LOC151760, LOC729911, long intergenic non-protein coding RNA 87 (LINC00087), HLA complex group 11 (HCG11), LOC100130987, LOC100507460, LOC338739, LOC100506631, C17orf76 antisense RNA 1 (C17orf76-AS1), chromosome 6 open reading frame 124 (C6orf124), LOC100509507, LOC100131606, ribosomal protein L13 pseudogene 5 (RPL13P5), CDC14 cell division cycle 14 homolog C (CDC14C), high mobility group protein B3-like (LOC646993), KRBOX1-AS1 (LOC100506275), long intergenic non-protein coding RNA 317 (LINC00317), LOC100293962, SAMD12 antisense RNA 1 (SAMD12-AS1)* and *kinesin family member 27 pseudogene (LOC389765)* were up-regulated versus the normal case (Supplementary 1 and Fig. 3).

### Protein class sorting

All non-obstructive azoospermia and normal cell groups were found to have differentially expressed transcripts (Table 1). Analysis of transcripts (using PANTHER) showed that differentially expressed RNAs cover diverse gene sequences localized all around the cell, including Molecular function: binding (GO:0005488), ATP-dependent activity (GO:0140657), catalytic activity (GO:0003824), cytoskeletal motor activity (GO:0003774), molecular function regulator (GO:0098772), molecular transducer activity (GO:0060089), structural molecule activity (GO:0005198), transcription regulator activity (GO:0140110), transporter activity (GO:0005215) and Biological process: biological adhesion (GO:0022610), biological regulation (GO:0065007), cellular process (GO:0009987), developmental process (GO:0032502), growth (GO:0040007), Immune system process (GO:0002376), Localization (GO:0051179), metabolic process (GO:0008152), response to stimulus (GO:0050896), signaling (GO:0023052), reproduction (GO:0000003) (Fig. 1). In Table 1, we present these biological processes and molecular functions along with the genes involved and their percentage involvement.

**Fig. 4:**
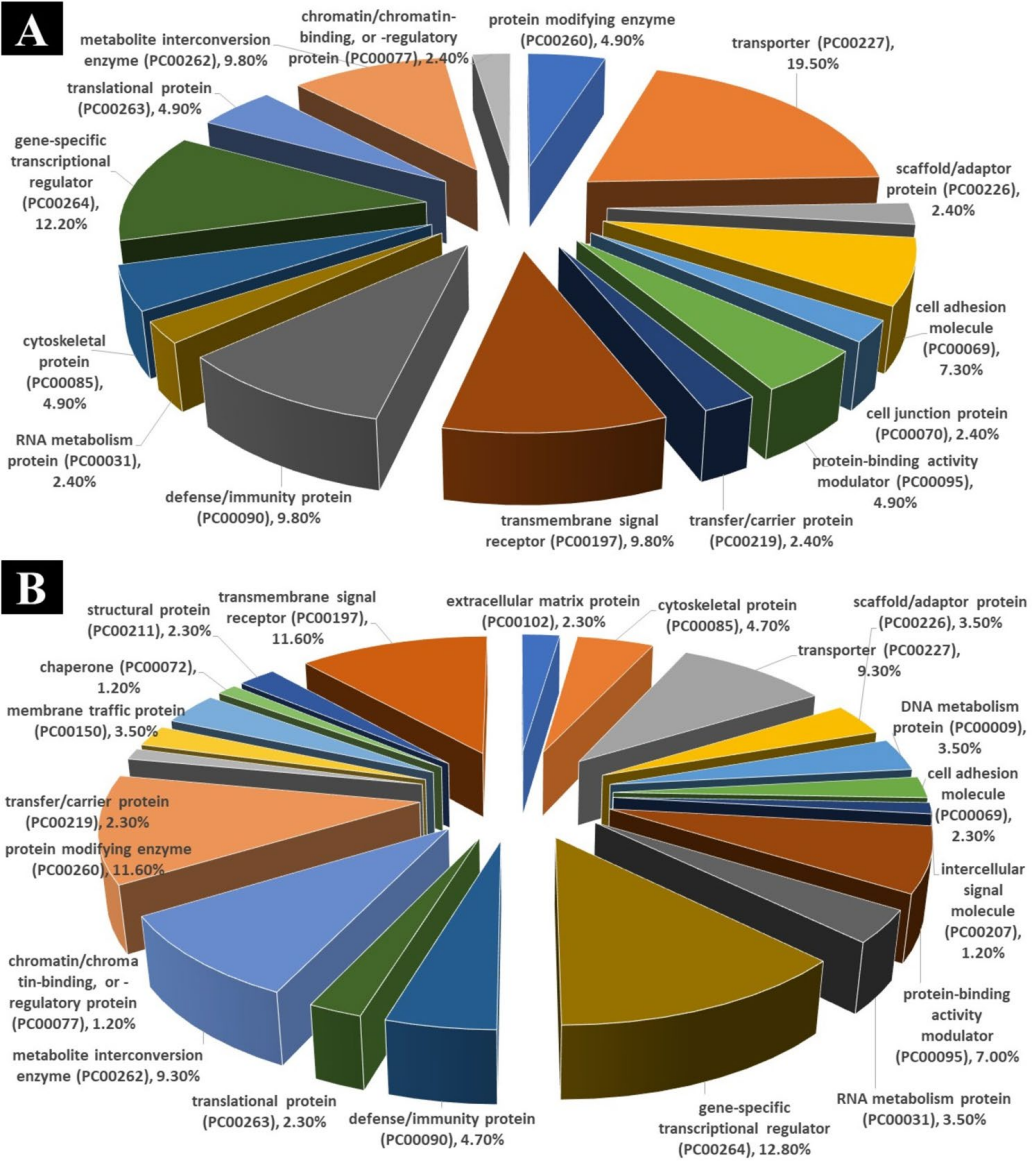
Analyzing protein classes and comparing groups. Using PANTHER, transcripts that differ significantly in expression are classified into protein classes based on their significant differences in expression

PANTHER server shows that down-regulation genes involved in protein modifying enzyme (PC00260) (4.9%), transporter (PC00227) (19.5%), scaffold/adaptor protein (PC00226) (2.4%), cell adhesion molecule (PC00069) (7.3%), cell junction protein (PC00070) (2.4%), protein-binding activity modulator (PC00095) (4.9%), transfer/carrier protein (PC00219) (2.4%), transmembrane signal receptor (PC00197) (9.8%), defense/immunity protein (PC00090) (9.8%) RNA metabolism protein (PC00031) (2.4%), cytoskeletal protein (PC00085) (4.9%), gene-specific transcriptional regulator (PC00264) (12.2%), translational protein (PC00263) (4.9%), metabolite interconversion enzyme (PC00262) (9.8%) and chromatin/chromatin-binding, or -regulatory protein (PC00077) (2.4%) (Fig. 4A).

And PANTHER server shows that up-regulation genes involved in extracellular matrix protein (PC00102) (2.3%), cytoskeletal protein (PC00085) (4.7%), transporter (PC00227) (9.3%), scaffold/adaptor protein (PC00226) (3.5%), DNA metabolism protein (PC00009) (3.5%), cell adhesion molecule (PC00069) (2.3%), intercellular signal molecule (PC00207) (1.2%), protein-binding activity modulator (PC00095) (7.0%), RNA metabolism protein (PC00031) (3.5%), gene-specific transcriptional regulator (PC00264) (12.8%), defense/immunity protein (PC00090) (4.7%), translational protein (PC00263) (2.3%), metabolite interconversion enzyme (PC00262) (9.3%), protein modifying enzyme (PC00260) (11.6%), chromatin/chromatin-binding, or -regulatory protein (PC00077) (1.2%), transfer/carrier protein (PC00219) (2.3%), membrane traffic protein (PC00150) (3.5%), transmembrane signal receptor (PC00197) (11.6%), structural protein (PC00211) (2.3%) and chaperone (PC00072) (1.2%)(Fig. 4B).

### The biological process of enrichment analysis and its molecular functions are as follows

**Fig. 5:**
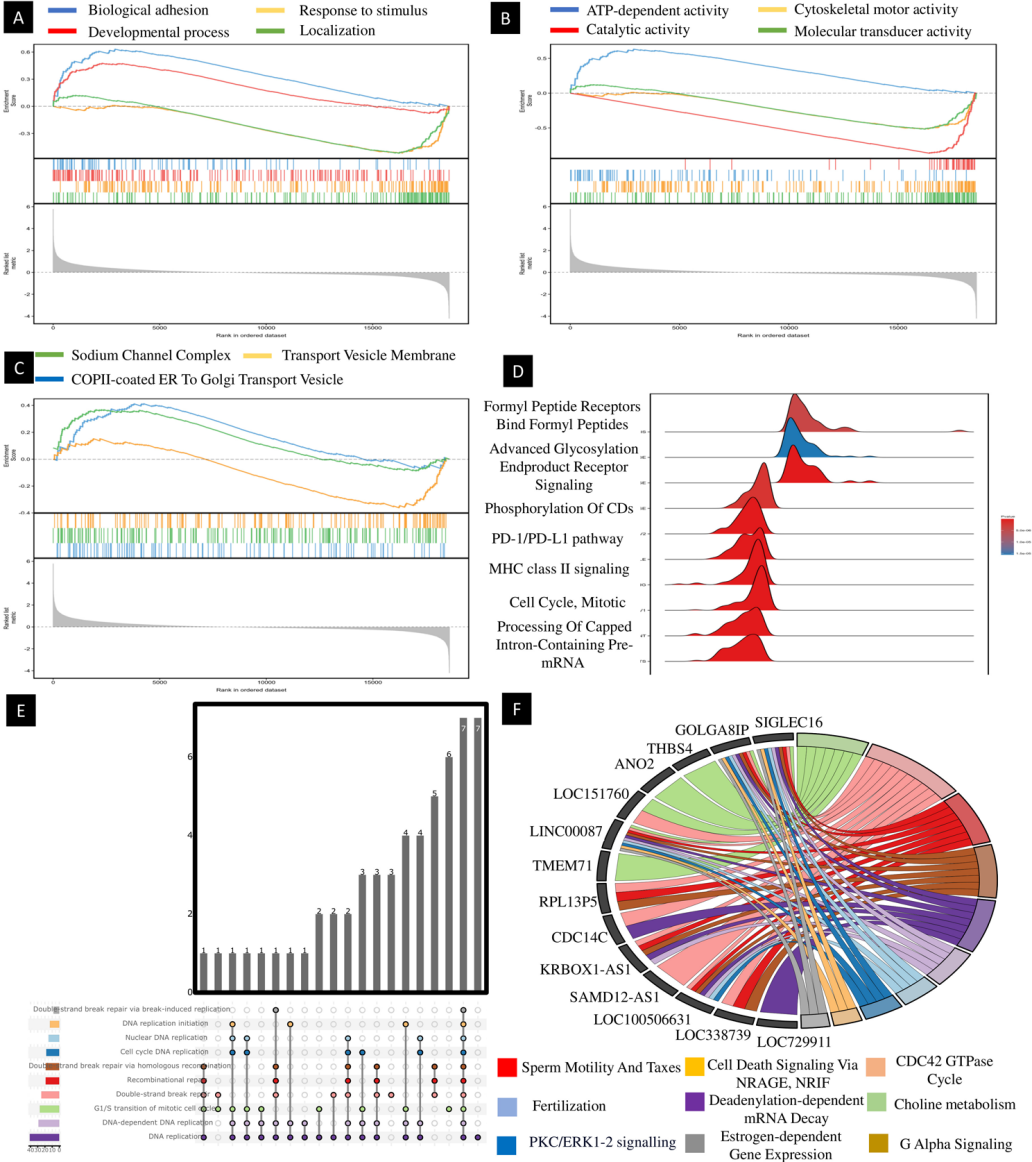
Based on Enrich and ShinyGO, we analyze biological processes, molecular functions, cellular component, and signallimg pathways in gene and ncRNA. (**A**) biological processes analysis, (**B**) moleculare function analysis, (**C**) cellular component, (**D**) signalling pathway analysis, (**E**) moleculare function analysis on ncRNA and (**F**) signalling pathway on ncRNA

In enrich tool analysis, three GO terms were incorporated with up-regulated DEGs, while three GO terms were incorporated with down-regulated DEGs. Functional enrichment analysis demonstrated that the biological process (BP) term “protein localization to endosome” (GO:0036010) (*p* < 0.001), “regulation of mitochondrial fission” (GO:0090140)(*p* < 0.0001), “xenobiotic transport” (GO:0042908) (*p* < 0.0001), “mitochondrial fission” (GO:0000266) (*p* < 0.0001), “protein exit from endoplasmic reticulum” (GO:0032527) (*p* < 0.0001) and “sodium ion transmembrane transporter” (GO:0015081) (*p* < 0.0001) was significantly overexpressed in up-regulated DEGs (Fig. 5a).

BP investigation of down-regulated DEGs highlighted “chloride transmembrane transport” (GO:1902476) (p < 0.001), “inorganic anion transmembrane transport” (GO:0098661) (p < 0.002), “inorganic anion transport” (GO:0015698) (p < 0.004), and “establishment of organelle localization” (GO:0051656) (p < 0.006) (Fig. 5b).

Overrepresented molecular function (MF) terms in up-regulated DEGs included “voltage-gated calcium channel activity” (GO:0005245) (*p* < 0.0001), “growth hormone-releasing hormone receptor activity” (GO:0016520) (*p* < 0.001), “sialic acid transmembrane transporter activity” (GO:0015136) (*p* < 0.001), “urate transmembrane transporter activity” (GO:0015143) (*p* < 0.001) and “glycerol channel activity” (GO:0015254) (*p* < 0.001) (Fig. 5c). Interestingly, MF analysis of down-regulated DEGs revealed an overexpression in “smoothened binding” (GO:0005119) (*p* < 0.001), " dATP binding” (GO:0032564) (*p* < 0.001) and “carnitine O-palmitoyltransferase activity” (GO:0004095) (*p* < 0.001) (Fig. 5). Figure 5 show that up/ down-regulation genes involved some important GO biological process, molecular function, and signaling pathways.

### Identification of key DEGs through PPI analysis

**Fig. 6:**
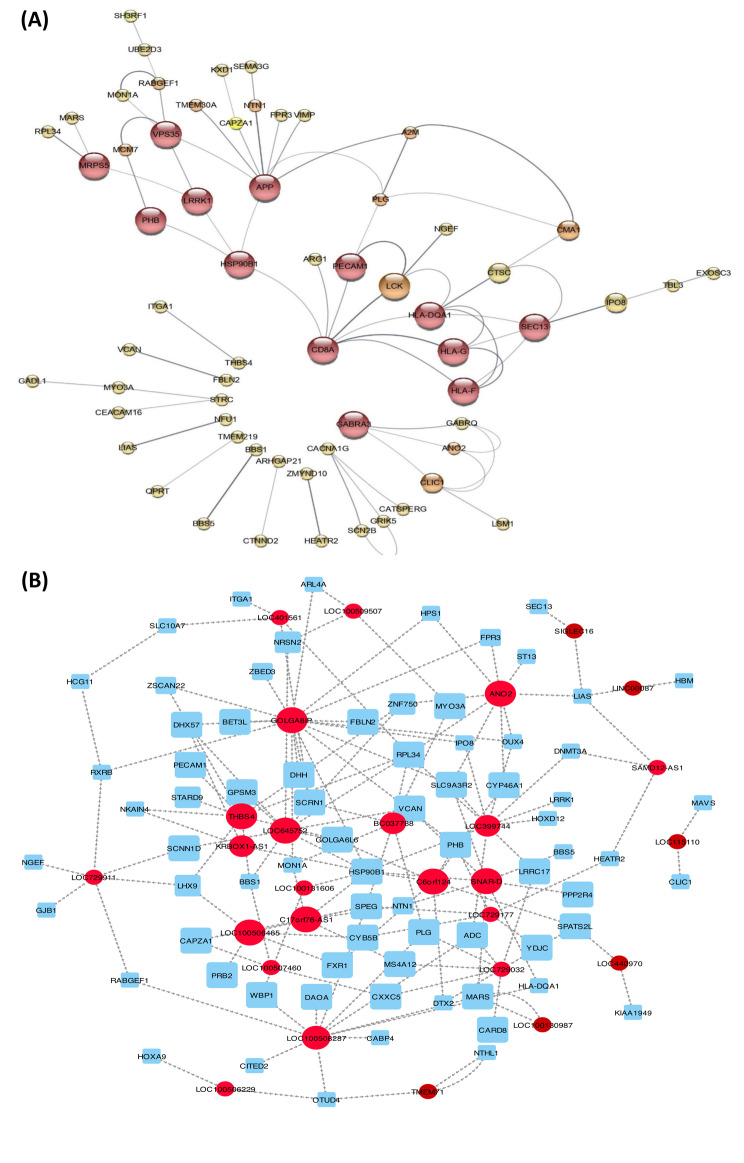
Based on gene interaction, STRING proteins interact with proteins and lncRNA–mRNA co-expression networks of some important nodes for comparison between Sertoli of NOA and health cell. (**A**) Up-/down-regulation of PPI networks and associated genes, Direct linkage between genes correlated with up-/down-regulation. (**B**) The genes and lncRNAs with |DiffK|>0.7 are presented in supplementary 2. indirect lncRNA–mRNA co-expression inactions, direct lncRNA–mRNA co-expression inactions and, protein-protein interaction based on co-expression and closeness in DEG mRNA

The master genes were identified using Cytoscape’s network analysis. In Fig. 6, yfiles redial layout was used to upload the highlighted genes in Fig. 4. Based on interactions between DEGs, the network classified them. As shown in the illustration, the 85 genes with the highest interaction have the highest correlation. A number of genes are correlated with *CD8A, GABRA3, HSP90BA, PECAM1, HLA-G*, and *Sect. 13*, and they are the only and primary regulators of these genes. In the other layouts of the Cytoscape network, high significant genes were found to interact. In order to verify the yfiles redial design, the Cytoscape network analysis tool was used. The Centiscape plugin represented *SPHAR, ADAM33, ACER2, MYO19, SLC17A3, EPC2, FBLN2, KRTAP20-3, SNORD114-28, SMCP, RABGEF1, SFSWAP, SH3RF1, ARHGAP33, APP, CCDC8, RNPEP, LRRC17, NTHL1, MYO3A, SCAF11, HLA-F, ACTR3B, CORIN, ZNF556* and *lLUZP1*, as the most important hub genes based on degree and betweenness centrality (Fig. 6a). With Cytoscape, the PPI network graph was visually analyzed based on the STRING database. By filtering out up/down regulation genes using the CytoHubba plug-in, 50 differential genes in the gene regulatory network were intersected, resulting in 14 hub target genes - *CDBA, HSP90B1, PECAM1, HLA-QA1, HLA-G, HLA-F, GABRA3, Sect. 13, APP, LRRK1, VDS35*, and *MRPS5*. In Fig. 6, *CDBA* is identified as the core target gene and exists in the gene regulatory network based on the analysis of differentially expressed genes. A gene regulatory network analysis identified *LCK, CMA1, CLIC1, RABGEF1, MCM7*, and *CACNA1G* as correlates and neighborhood genes.

### lncRNA–mRNA co-expression network

Using sperm of NOA as a model, we explored the intersecting genes by selecting shared genes for significantly enhanced functions and pathways. There were 196 genes that intersected between the two groups. NOA Sertoli cells and normal Sertoli cells were selected to identify differentially expressed lncRNAs and intersecting genes. A lncRNA-mRNA co-expression network was constructed by determining how lncRNAs and intersecting genes interact and are regulated. Among 214 genes (mRNAs) and 35 corresponding lncRNAs in the lncRNA–mRNA co-expression network, 194 interactions have been identified. An overview of some of the most significant nodes in the lncRNA–mRNA co-expression network is presented in Fig. 6B and supplementary 2. We identified crucial genes and lncRNAs that determine essential distinctions by using differential co-expression networks between lncRNA and mRNA. Figure 6B shows gene and lncRNA pairs with |DiffK|>0.7.

### NOA versus normal gene expression profiles based on co-expression analysis and confirm by microarray

There are several nodes with high degrees and clustering coefficients that indicate extraordinary expression (DiffK > 0.7), including *PHB, LHX9, OTUD4, SPATS2L, LIAS, RXRB, SCRN1, PHB, LHX9, OTUD4, SPATS2L, LIAS, RXRB, SCRN1, PHB, LHX9* and *ITGA1*. As shown in Fig. 4; Table 3, the lncRNA–mRNA co-expression network of some of the most important nodes is a network of lncRNA–mRNA co-expression.

**Table 3:** GO, Gene involved, and percentage GO in up-regulation genes

UP regulation	GO	Gene involved	percentage
Molecular function	binding (GO:0005488)	GHRHR, CYP46A1, ACBD4, DHX57, MTUS1, HOXA9, FOXO6, PCYT1A, ONECUT3, ZNF519, MAGEA8, LCK, HLA-DQA1, APP, HOXD12, HPS1, ACTR3B, RXRB, ZNF556, GRIK5, ARL4A, RABGEF1, PAX3, ZSCAN22, HSP90B1, LTB4R2, DHH, ARHGAP33, MCM7, FPR3	45%
	ATP-dependent activity (GO:0140657)	MCM7, MYO19	2%
	catalytic activity (GO:0003824)	CYP46A1, PCYT1A, SH3RF1, LCK, QPRT, RNPEP, HPS1, RABGEF1, NTHL1, DTX2, ARHGAP33, MCM7, PLG, STK4, ARG1, OTUD4, PIGO, MYO19, MOXD1, CDC14C	20%
	cytoskeletal motor activity (GO:0003774)	MYO19	1%
	molecular function regulator (GO:0098772)	APP, HPS1, RABGEF1, ARHGAP33, SEMA3G	5%
	molecular transducer activity (GO:0060089)	GHRHR, OR6C65, APP, RXRB, GRIK5, LTB4R2, FPR3, PECAM1, SEMA3G	9%
	structural molecule activity (GO:0005198)	MRPS5	1%
	transcription regulator activity (GO:0140110)	HOXA9, FOXO6, ONECUT3, ZNF519, RXRB, ZNF556, PAX3, ZSCAN22, LHX9	9%
	transporter activity (GO:0005215)	FAM155B, SCNN1D, AQP7, SLC35E1, GRIK5, SLC17A3, CACNA1G, CLIC1	8%
Biological process	biological adhesion (GO:0022610)	HLA-DQA1, STRC, PECAM1, CTNND2	4%
	biological regulation (GO:0065007)	GHRHR, FAM155B, HOXA9, FOXO6, EPC2, SH3RF1, ONECUT3, SELS, ZNF519, MAGEA8, LCK, MS4A12, HLA-DQA1, TMEM30A, RXRB, ZNF556, GRIK5, NKAIN4, PAX3, ZSCAN22, DTX2, LTB4R2, DHH, ARHGAP33, FPR3, CACNA1G, FXR1, PECAM1, STK4, EAPP,	18%
	cellular process (GO:0009987)	GHRHR, FAM155B, ACBD4, PHB, HOXA9, FOXO6, EPC2, GOLGA8IP, SH3RF1, SCNN1D, ONECUT3, SELS, ZNF519, MAGEA8, SFRS8, LCK, MS4A12, HLA-DQA1, TMEM30A, QPRT, APP, ACTR3B, RXRB, ZNF556, GRIK5, ARL4A, C2orf65, NTHL1, PAX3, ZSCAN22	20%
	developmental process (GO:0032502)	LCK, HOXA9, APP, RXRB, C2orf65, PAX3, DHH, FXR1, LHX9, CTNND2, SEMA3G	8%
	growth (GO:0040007)	SEMA3G	1%
	Immune system process (GO:0002376)	HLA-DQA1, FPR3, OTUD4, HLA-F, LCK	4%
	Localization (GO:0051179)	SCNN1D, SELS, AQP7, TMEM30A, ARL4A, NKAIN4, SLC17A3, CACNA1G, FXR1, VPS35, Sect. 13, CLIC1, TAPT1, SEMA3G, EXOSC3, MYO19, CDC14C	10%
	locomotion (GO:0040011)	SEMA3G	1%
	metabolic process (GO:0008152)	CYP46A1, ACBD4, HOXA9, FOXO6, EPC2, SH3RF1, ONECUT3, SELS, ZNF519, MAGEA8, SFRS8, HLA-DQA1, QPRT, RNPEP, RXRB, ZNF556, NTHL1, PAX3, ZSCAN22, DTX2, HSP90B1, MRPS5, DHH, MCM7, PLG, FXR1, STK4, DNMT3A, SFRS2IP, ARG1	15%
	multicellular organismal process (GO:0032501)	LCK, HOXA9, APP, C2orf65, FXR1, LHX9, CTNND2, SEMA3G,	5%
	response to stimulus (GO:0050896)	SH3RF1, SELS, LCK, MS4A12, HLA-DQA1, RXRB, NTHL1, DTX2, HSP90B1, LTB4R2, DHH, ARHGAP33, MCM7, FPR3, FXR1, PECAM1, STK4, OTUD4, HLA-F, SEMA3G, HLA-DQA1	10%
	signaling (GO:0023052)	SH3RF1, SELS, LCK, MS4A12, RXRB, GRIK5, DTX2, LTB4R2, DHH, ARHGAP33, FPR3, FXR1, PECAM1 STK4, OTUD4, SEMA3G	8%
	reproduction (GO:0000003)	C2orf65	1%
	biological process involved in interspecies interaction between organisms (GO:0044419)	LCK	1%

### Biological process and molecular functions of enrichment analysis

**Fig. 7:**
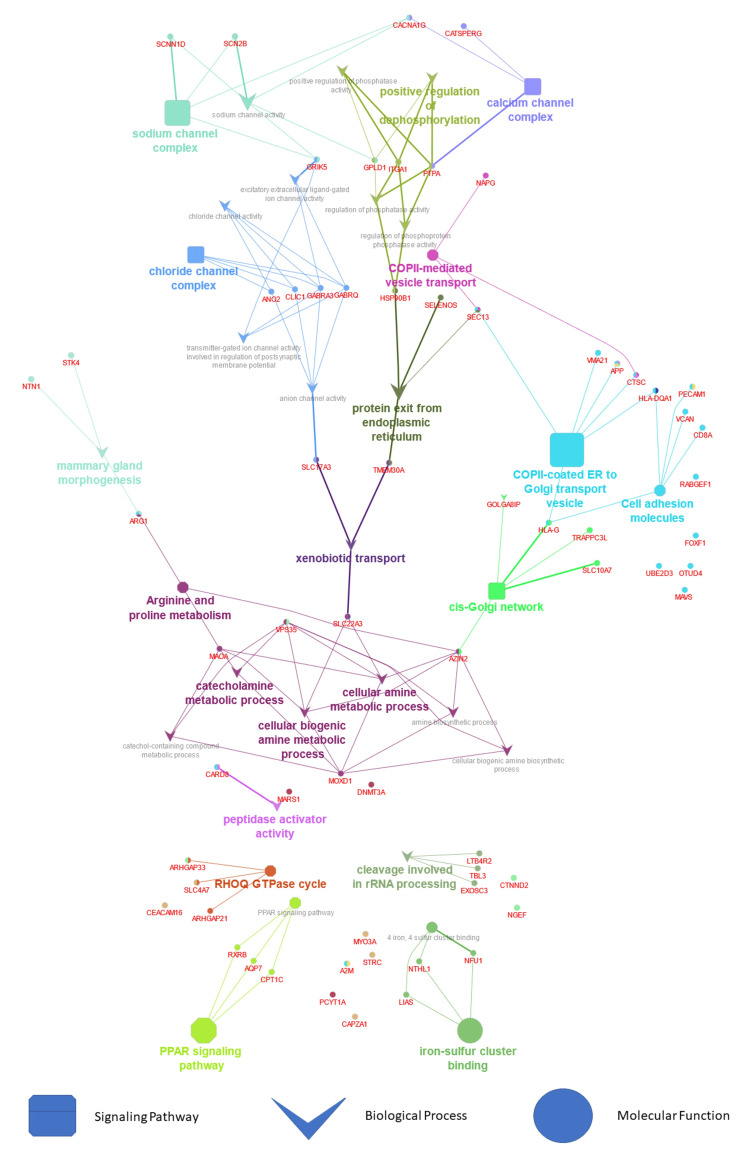
Signalling pathway analysis in gene

Genes and gene products are classified and described using GO analysis. Three domains were covered in the ontology: Biological Process (BP) and Molecular Function (MF). According to the enrich tool analysis, DEGs that were up-regulated were enriched in 3 GO terms, while DEGs that were down-regulated were enriched in 4 GO terms. In up-regulated DEGs (Fig. 5a), functional enrichment analysis revealed significant overexpressions of CD8-positive, alpha-beta cytotoxic T cells, Protein K6-linked deubiquitination, Regulation of tumor necrosis factor-mediated signaling pathways, and Regulation of necroptotic pathways. BP analysis of down-regulated DEGs showed JUN kinase activity, Ribonucleotide-diphosphate reductase activity, thioredoxin disulfide as acceptor, oxidoreductase activity, acting on CH or CH2 groups, disulfide as acceptor, tubulin deacetylase activity, and hemimethylated DNA binding (Fig. 7).

There was an overrepresentation of molecular function (MF) terms in up-regulated DEGs including Death effector domain binding, Inositol-1,3,4,5-tetrakisphosphate 5-phosphatase activity, Inositol-polyphosphate 5-phosphatase activity, Clathrin light chain binding, and Phosphatidylinositol-3,4,5-trisphosphate 5-phosphatase activity (Fig. 7). Mitotic Cytokinesis, Mitotic Spindle organization, Cytokinesis, Sister Chromatid segregation, and Microtubule cytoskeleton organization were all overexpressed in down-regulated DEGs (Fig. 7; Tables 3 and 4).

**Table 4:** GO, Gene involved, and percentage GO in down-regulation genes

Down regulation	GO	Gene involved	percentage
Molecular function	binding (GO:0005488)	GABRQ, SIGLEC16, BBS5, SLC9A3R2, HLA-G, NTN1, PIRT, HBM, BBS1, CARD8, ST13, ITGA1, LSM1, ZNF750, ZBED3, CAPZA1, PRB2, GABRA3, ASAP3, CXXC5, FOXF1, MARS, GPR150, CYB5B, CEBPE	50%
	catalytic activity (GO:0003824)	ENTPD2, HBM, CARD8, CTSC, ASAP3, SPEG, MARS, ADC, CPT1C, UBE2D3	22%
	molecular adaptor activity (GO:0060090)	SLC9A3R2	2%
	molecular function regulator (GO:0098772)	CARD8, ASAP3, MARS	6%
	molecular transducer activity (GO:0060089)	GABRQ, GABRA3, GPR150	6%
	transcription regulator activity (GO:0140110)	NTN1, ZNF750, FOXF1, CEBPE	8%
	transporter activity (GO:0005215)	SLC4A7, GABRA3, ANO2	6%
Biological process	biological adhesion (GO:0022610)	SIRPG, SIGLEC16, ITGA1	5%
	biological regulation (GO:0065007)	GABRQ, SIRPG, HLA-G, NTN1, PIRT, CARD8, ITGA1, LSM1, ZNF750, ZBED3, CAPZA1, PRB2, SLC4A7, GABRA3, NGEF, FOXF1, GPR150, CEBPE	30%
	cellular process (GO:0009987)	GABRQ, SIRPG, SIGLEC16, ENTPD2, BET3L, BBS5, SLC9A3R2, HLA-G, IPO8, NTN1, PIRT, HBM, BBS1, CARD8, ST13, ITGA1, LSM1, ZNF750, ZBED3, CAPZA1, PRB2, SLC4A7, GABRA3, CTSC, ANO2, FOXF1, MARS, GPR150, CPT1C	52%
	developmental process (GO:0032502)	NRSN2, ZNF750, PRB2, FOXF1, CEBPE	8%
	immune system process (GO:0002376)	SIRPG, HLA-G, CEBPE	5%
	localization (GO:0051179)	GABRQ, SIRPG, BET3L, BBS5, SLC9A3R2, IPO8, PIRT, BBS1, SLC4A7, GABRA3, ANO2	18%
	metabolic process (GO:0008152)	ENTPD2, NTN1, HBM, CARD8, LSM1, ZNF750, ZBED3, CTSC, FOXF1, MARS, CPT1C, UBE2D3, CEBPE	23%
	multicellular organismal process (GO:0032501)	NRSN2, GABRQ, GABRA3, FOXF1, CEBPE	8%
	response to stimulus (GO:0050896)	GABRQ, HLA-G, PIRT, CARD8, ITGA1, GABRA3, GPR150	12%
	signaling (GO:0023052)	GABRQ, PIRT, CARD8, ITGA1, GABRA3, GPR150	10

### Gene co-expression modules for signaling pathway

The DEGs between NOA and control were included in the WGCNA analysis was determined as soft-thresholding to acquire DEG co-expressed gene modules (Fig. 7A). Ultimately, DEGs were clustered into red, dark brown, green, and light brown modules, respectively. Among these gene modules, red module was significantly RHOQ GTPase cycle. Dark brown module was remarkably correlated with cellular amine metabolic process and green module was involved in PPAR signaling pathway. Light brown modules were significantly involved in cell adhesion molecules and chloride channel complex. Taken together, these two modules were positively or negatively relevant to the most growth hormone-releasing hormone receptor activity and sialic acid transmembrane transporter activity and were considered important transcriptionally dysregulated gene modules with regard to the altered gene expression.

### Identification of a hub gene from scRNA-seq

**Fig. 8:**
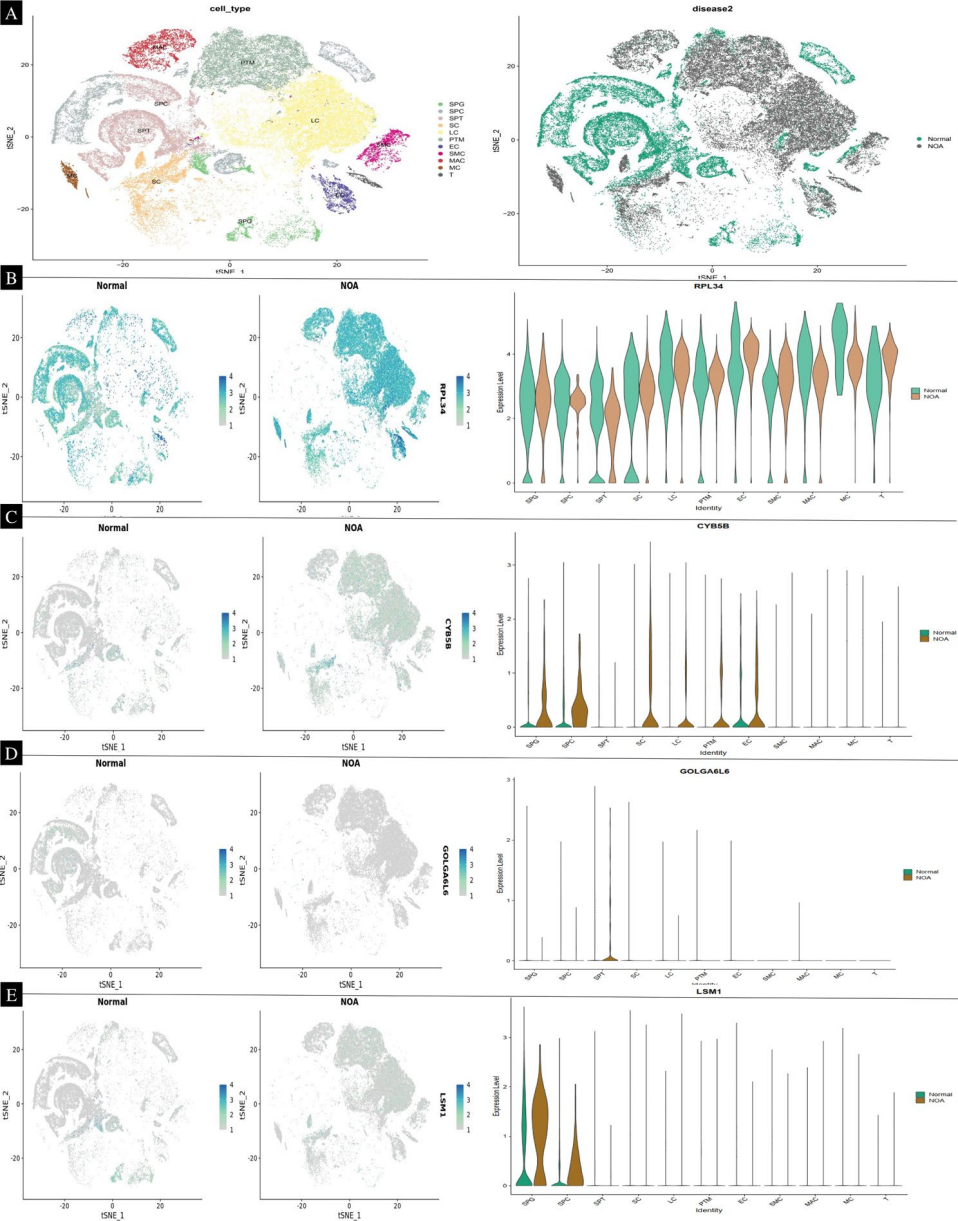
Identification of a NOA-specific hub gene in single cell. (**A**) The 29 samples were split into eleven clusters. The analysis of 21 normal and 7 non-obstructive azoospermia (NOA) samples (refer to Supplementary 3) revealed four distinct clusters, highlighting the diversity of cell types involved in spermatogenesis. These clusters included spermatogonia (SPG), spermatids/sperm (SPT), spermatocytes (SPS), Sertoli cells (SC), Leydig cells (LC), peritubular myoid cells (PTM), endothelial cells (EC), vascular smooth muscle cells (SMC), macrophages (MAC), mast cells (MC), and T-cells (T). Notably, this clustering demonstrated not only the transcriptomic differences between normal and NOA samples but also the intrinsic heterogeneity within the NOA samples. Single-cell transcriptomic analysis identified nine hub genes - RPL34, CYB5B, GOL6A6, LSM1, ARL4A, DHX57, STARD9, HSP90B1, and VPS36 - as significantly associated with NOA sperm. Among these genes, RPL34, CYB5B, GOL6A6, and LSM1 were upregulated in NOA samples. In contrast, ARL4A, DHX57, STARD9, HSP90B1, and VPS36 were found to be downregulated, suggesting their potential roles in the pathogenesis of NOA

**Fig. 9:**
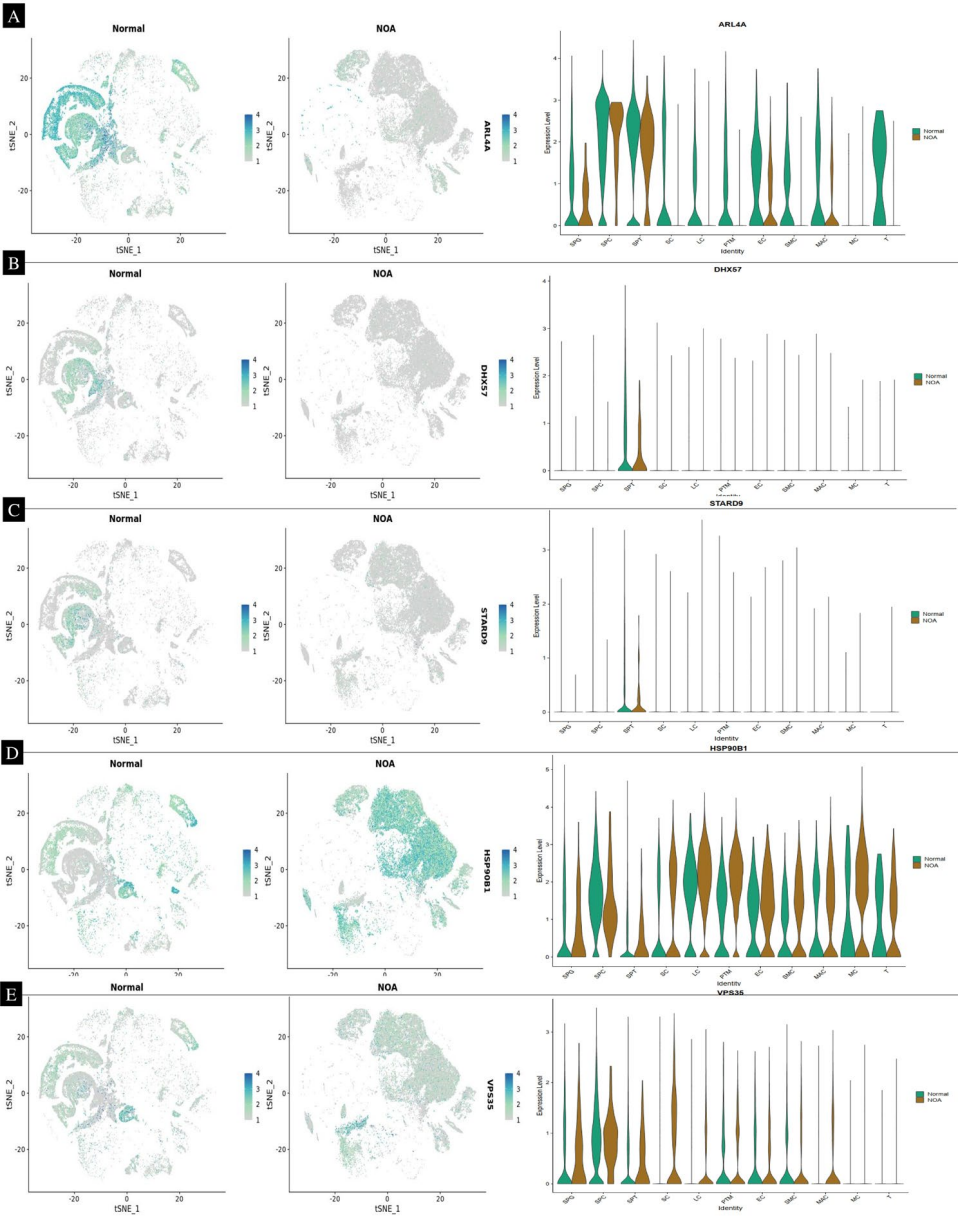
ARL4A, DHX57, STARD9, HSP90B1, and VPS36 were found to be downregulated, suggesting their potential roles in the pathogenesis of NOA

In our study, we analyzed gene expression data from 29 samples in the GSE45885 dataset, which included 3 samples from normal individuals and 26 from non-obstructive azoospermia (NOA) patients. To explore the heterogeneity in NOA, we performed unsupervised clustering on a subset of 28 samples, comprising 21 normal and 7 NOA samples (see Supplementary 3). This analysis yielded four distinct clusters. These clusters encompassed various cell types relevant to spermatogenesis, such as spermatogonia (SPG), spermatids/sperm (SPT), spermatocytes (SPS), Sertoli cells (SC), Leydig cells (LC), peritubular myoid cells (PTM), endothelial cells (EC), vascular smooth muscle cells (SMC), macrophages (MAC), mast cells (MC), and T-cells (T). This clustering not only highlighted transcriptomic differences between normal and NOA samples but also underscored the heterogeneity within the NOA samples themselves. Further analysis using principal component analysis (PCA) reinforced these findings (refer to Figs. 8 and 9). Additionally, our single-cell data analysis identified nine hub genes – RPL34, CYB5B, GOL6A6, LSM1, ARL4A, DHX57, STARD9, HSP90B1, and VPS36 – that are significantly associated with NOA sperm. Among these, RPL34, CYB5B, GOL6A6, and LSM1 were found to be upregulated in NOA samples (Fig. 8), whereas ARL4A, DHX57, STARD9, HSP90B1, and VPS36 showed downregulation (Fig. 9).

### The following candidate microRNAs have been isolated and selected

**Fig. 10:**
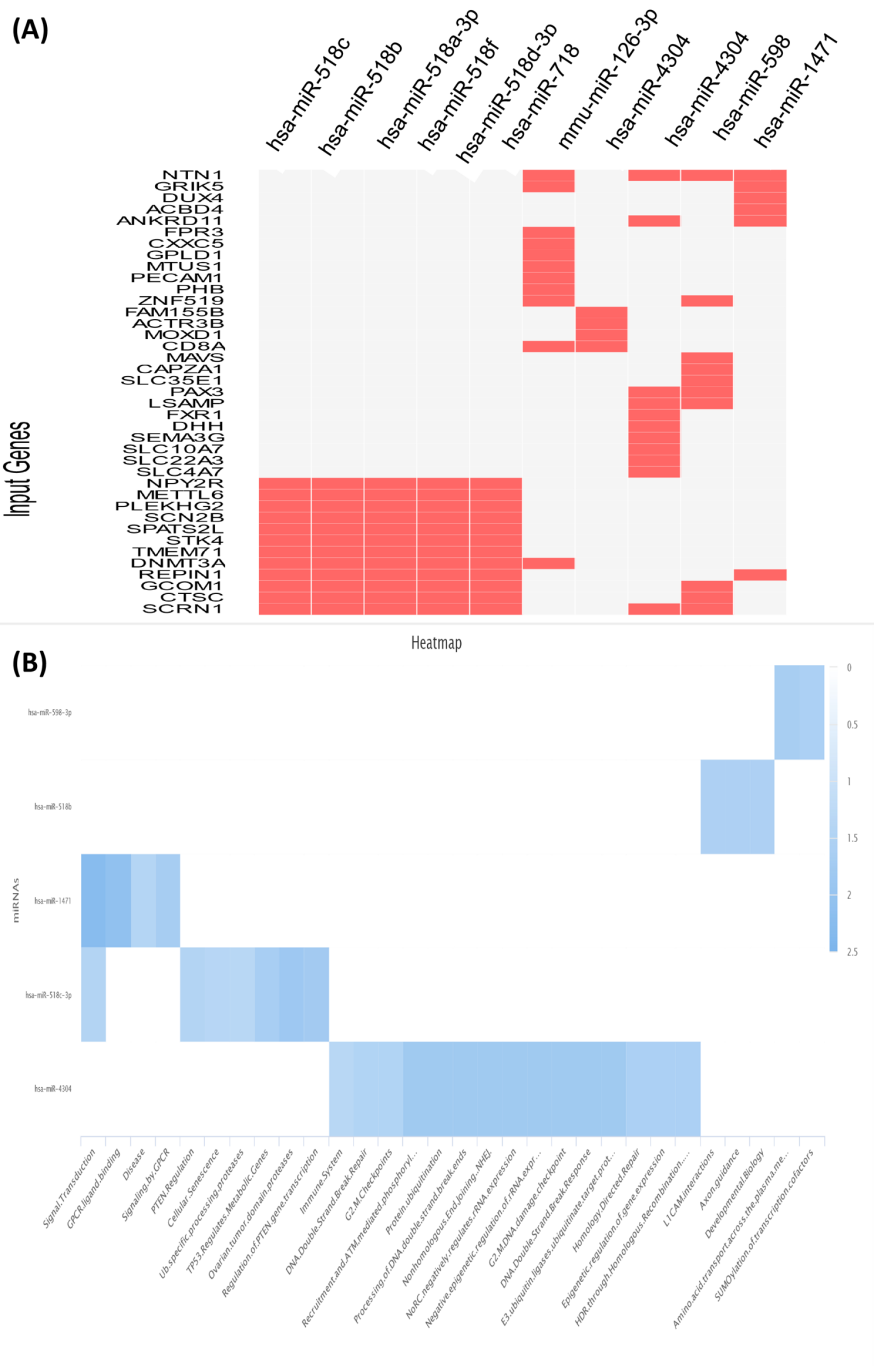
Hub genes and miRNAs that target hub genes. In the miRNA-gene regulatory network, nine differential miRNAs were intersected with up/down-regulated genes

In this section, after identifying 15 genes, *SCRN1, CTSC, GCOM1, REP1N1, DNMT3A, STK4, SPTS2L, SCN2B, PLEKHG2, METTL6, NPY2R, NTN1, GRIK5, ANKRD11, ZNF519* and *CD8A*, we isolated and selected the most relevant microRNAs (Table 5). Accordingly, hsa-miR-518c, hsa-miR-518b, hsa-miR-518a-3p, hsa-miR-518f, hsa-miR-518d-3p, hsa-miR-718, mmu miR-126-3p, hsa-miR-4304, hsa-miR-598 and hsa-miR-1471 were observed more clearly than other microRNAs (Fig. 10). These microRNAs are candidates for up and down-regulation of SCRN1, CTSC, GCOM1, REP1N1, DNMT3A, STK4, and SPTS2L genes (Fig. 10; Table 5).

**Table 5:** P-value, q-value, and overlap gene with candidate microRNAs extracted in miRNA databases

Name	*P*-value	Adjusted *p*-value	Odds Ratio	Combined score
hsa-miR-518c	0.02426	1.000	1.98	7.38
hsa-miR-518b	0.02426	1.000	1.98	7.38
hsa-miR-518a-3p	0.02426	1.000	1.98	7.38
hsa-miR-518f	0.02426	1.000	1.98	7.38
hsa-miR-518d-3p	0.02426	1.000	1.98	7.38
hsa-miR-718	0.02984	1.000	1.99	6.97
mmu-miR-126-3p	0.08472	1.000	2.49	6.14
hsa-miR-4304	0.06322	1.000	1.74	4.80
hsa-miR-598	0.09010	1.000	1.67	4.01
hsa-miR-1471	0.1613	1.000	1.67	3.05

## Discussion

The aim of this article is to provide an overview of all the genetic defects known to be linked to NOA in humans and identify genes and noncoding RNAs. We use some databases online to predict some GO, signaling pathways, and miRNA involved in these processes. Cytoskeletal elements are required for spermatogenic cells to alter morphologically and translocate in the seminiferous tubule during spermatogenesis. Actin filaments are found in certain locations of spermatogenic cells and are controlled by various actin-binding proteins [63]. One of the crucial actins regulating proteins is actin capping protein, and recent research found that testis-specific actin capping protein may impact male infertility [64, 65]. Actin filaments are involved in several critical stages of spermatogenesis, including acrosome biogenesis, flagellum creation, nuclear processes, and complex synaptonemal formation [66, 67]. Our microarray analysis revealed that *KRT35, ACTR3B, CTNND2, and MYO19, were up-regulated, while CAPZA1* and *GOLGA6L6* were down-regulated. We hypothesized that altering these cytoskeletal elements’ gene expression may change morphologically and translocate in the sperm.

In Sertoli-spermatid junctions, the cadherin-catenin system is found. Whether cadherin-catenin is necessary for Sertoli-spermatid junction development is unclear [68]. Sertoli-spermatid junctions are now known to be dependent on the nectin-afadin system [69]. In forming Sertoli-spermatid junctions, heterophilic trans-interaction of nectin-2 and nectin-3 creates cell adhesion, then recruits N-cadherin to the adhesion sites based on nectin, and finally establishes strong adhesion undercoated with F-actin mediated by afadin and catenins. *THBS4 and STRC were upregulated, whereas GJB1, SIGLEC16, ITGA1*, and *CEACAM16* were downregulated. A cell polarity complex cannot be assembled without these genes. We hypothesized that altering the expression of these genes may affect polarity protein complex formation and spermatid development.

A large amount of DNA methylation is involved in the early stages of spermatogenesis. This persists throughout meiosis and subsequent gamete development, impacting only a few genes at a time [70]. This is the epigenetic reprogramming step, in which each cell produces the appropriate [71]. After fertilization, so-called “imprinted genes” escape epigenetic reprogramming, allowing sperm abnormal DNA methylation to persist in the developing embryo [72]. The preservation of parent-specific germline patterns may support the participation of epigenetic flyws in possible offspring risk during ART. Several studies have shown a substantial relationship between DNA methylation changes of particular imprinted genes and spermatozoa in males with idiopathic infertility. When infertile males were compared to fertile men, an elevated odds ratio of imprinting abnormalities in two particularly imprinted genes, H19 and mesoderm-specific transcript (MEST), was found [73, 74]. Our microarray analysis showed that *NTHL1, MCM7, DNMT3A, DHX57, SFRS8*, and *EXOSC3* were up-regulated, while *LSM1* was down-regulated. These genes are necessary for DNA/ RNA metabolism protein. We hypothesized that altering the expression of these genes may change the polarity protein complex formation and spermatid development.

Ion channels control membrane potential and intracellular ionic concentration and so play an important role in many cellular activities [75]. Several ion channels, notably sperm, have been found in germ cells, indicating their relevance in male fertility and reproduction. Although multiple ligand-gated and voltage-gated channels have been found and localized on sperm, the molecular mechanism of ion transport and the type of the ion channels involved have only lately begun to emerge [76–78]. Our microarray analysis showed that *FAM155B, SCNN1D, AQP7, SLC35E1, GRIK5, SLC17A3, CACNA1G*, and *CLIC1* were up-regulated, while *SLC4A7, GABRA3* and *ANO2* were down-regulated.

Male reproductive system malfunctions are caused by the spermatogenic arrest. The production of spermatozoa is prevented by a wide variety of ncRNAs that disrupt the differentiation of different spermatogenic cell types throughout this process [79–81]. Specifically, knocking down testis-specific lncRNA 1 (Tslrn1) rendered mice sterile when their testes were knocked down. In this study, aberrant expression of lncRNA might have an impact on male reproduction. Mice’s ncRNA-Tsx gene is located near the X-inactivation site and is expressed by germ and stem cells. Although the kids survive, the suppression of this gene leads in reduced testes and aberrant X-inactivation sites in stem cells. There are many ncRNAs found in human sperm that have been associated with spermatogenesis and male fertility, and many of them are tissue-specific. As compared to the normal case, 24 ncRNAs were up-regulated and 13 ncRNAs were down-regulated in three human cases with non-obstructive azoospermia. All of these ncRNAs were found in the sperm of infertile males and were shown to be connected, indicating that they may be linked to sperm viability. A team of researchers has found that some ncRNA golgi autoantigen, *golgin subfamily, LOC645752, LOC729032, FMO9P, LOC100130825, GOLGA8IP* and *small ILF3/NF90-hThis ncRNA* may serve as a new therapeutic target for sperm suffering from azoospermia.

Our study stands out due to its comprehensive approach to understanding NOA. Previous studies have primarily focused on identifying individual genetic markers or specific pathways involved in NOA. For instance, a notable study by Aston et al. [82] concentrated on the role of single-nucleotide polymorphisms in NOA patients, offering valuable insights into the genetic predispositions of this condition. However, our research extends beyond this by utilizing unsupervised clustering to unravel the complex heterogeneity inherent in NOA at a cellular level. This approach not only confirms the findings from earlier genetic studies but also provides a broader perspective on the varying transcriptomic landscapes within NOA samples.

Moreover, our identification of hub genes specifically associated with NOA adds a novel dimension to the existing literature. While earlier studies like that of Tomoiaga et al. relied on bulk RNA sequencing, our use of single-cell sequencing allows for the precise characterization of cell-specific gene expression profiles [83]. This advancement not only validates previous findings but also unveils new insights into the molecular mechanisms underlying NOA pathogenesis. Our study highlights the crucial role of cytoskeletal elements, cell adhesion molecules, and DNA methylation in spermatogenesis and male fertility. For instance, the upregulation of *KRT35, ACTR3B, CTNND2*, and *MYO19* suggests potential alterations in sperm morphology and function, while the downregulation of *CAPZA1* and *GOLGA6L6* implicates disruptions in cytoskeletal organization. Furthermore, the dysregulation of genes involved in cell adhesion complexes, such as *THBS4* and *STRC*, underscores their importance in Sertoli-spermatid junction development. Clinically, our findings hold promise for personalized approaches to male infertility diagnosis and treatment. By identifying specific genes and pathways associated with NOA, such as *FAM155B, SCNN1D*, and *AQP7*, we pave the way for targeted interventions tailored to individual genetic profiles. Furthermore, our comprehensive analysis underscores the potential of precision medicine in revolutionizing reproductive medicine and improving outcomes for patients with NOA.

## Conclusion

Based on a list of candidate genes and non-coding RNA, we used here a simple strategy that facilitates the interpretation of microarray data and the identification of causal gene defects with predictions of their consequences for spermatogenesis. Considering other clinical arguments, our results indicate that a clinical diagnosis against sperm can be very persuasive. Gene expression and scRNA-seq analysis can provide clinicians with more precise and relevant information that will allow them to provide their patients with the most appropriate course of action, possibly avoiding hopeless surgical procedures, in some instances.

### Electronic supplementary material

Below is the link to the electronic supplementary material.

Supplementary Material 1

Supplementary Material 2

Supplementary Material 3

## Data Availability

The microarray data have been deposited in GEO (https://www.ncbi.nlm.nih.gov/geo) under the accession number GSE268452, and supplementary data during the current study are available on the Zenodo website, https://zenodo.org/records/10673208, respectively.

## Abbreviations

ncRNA: Non-coding RNA
OA: Obstructive azoospermia
NOA: Non-obstructive azoospermia
SMCP: Sperm mitochondria-associated cysteine-rich protein
DNAJB13: DnaJ homolog subfamily B member 13
CREM: cAMP response element modulator
scRNA-seq: Single-cell RNA sequencing
scATAC-seq: Single-cell assay for transposase-accessible chromatin sequencing
FSH: Follicle-stimulating hormone
PCCs: Pearson’s correlation coefficients
WGCNA: Weighted Gene Co-Expression Network Analysis
Tslrn1: Testis-specific lncRNA 1
GO: Gene ontology
DEGs: Differentially Expressed Genes
MF: Molecular function
PPI: Protein-protein interaction
